# ¹H-NMR serum metabolomic profiling from clinical routine identifies signatures of progressive melanoma metastasis

**DOI:** 10.1038/s41598-026-37118-5

**Published:** 2026-01-30

**Authors:** Frank Friedrich Gellrich, Cosima Hufnagel, Alexander M. Funk, Sophie Jonas, Heidi Altmann, Sarah Hobelsberger, Julian Steininger, Ricarda Rauschenberg, Marlene Garzarolli, Sophia Lehr, Lea Pöschman, Paula Biedermann, Julia Sophie Schatz, Christina Feige, Alpaslan Tasdogan, Triantafyllos Chavakis, Stefan Beissert, Friedegund Meier, Peter Mirtschink, Gerald Steiner

**Affiliations:** 1 Department of Dermatology, Faculty of Medicine and University Hospital Carl Gustav Carus, Technische Universität Dresden, Dresden, Germany; 2 Skin Cancer Center at the University Cancer Center and National Center for Tumor Diseases Dresden, Dresden, Germany; 3 BioBank Dresden, Faculty of Medicine and University Hospital Carl Gustav Carus, Technische Universität Dresden, Dresden, Germany; 4 National Center for Tumor Diseases, Dresden (NCT/UCC), Dresden, Germany; 5 Department of Anesthesia and Intensive Care, Clinical Sensoring and Monitoring, Faculty of Medicine Carl Gustav Carus, University Hospital, Technische Universität Dresden, Dresden, Germany; 6 Institute for Clinical Chemistry and Laboratory Medicine, Technische Universität Dresden, 01307 Dresden, Germany; 7 Department of Dermatology, University Hospital Essen & German Cancer Consortium (DKTK), Partner Site Essen, Essen, Germany; 8 National Center for Tumour diseases (NCT-West), Campus Essen & Research Alliance Ruhr,, Research Center One Health, University Duisburg-Essen, Essen, Germany

**Keywords:** Biomarkers, Metabolic profiling, Melanoma, Tumor metabolism, Predictive model, Biomarkers, Cancer, Oncology

## Abstract

Early detection of active melanoma metastasis is crucial. Serum metabolomics may offer non-invasive biomarkers, but real-world applicability needs validation. This study aimed to identify ¹H-NMR-based serum metabolic signatures for active metastasis in a large clinical cohort. Serum from 963 melanoma patients (1698 samples) underwent ¹H-NMR spectroscopy. Patients were classified by active metastasis status. OPLS-DA and RFE followed by logistic regression models were developed on a patient-level training/test split. Subgroup analyses assessed signatures related to Immune Checkpoint Inhibitor (ICI) therapy, brain metastases, and BRAF status. Models for active metastasis showed moderate test set discrimination (Area Under the Curve [AUCs]: OPLS-DA 0.609, RFE 0.630). The RFE-model highlighted seven significant metabolites: increased pyruvate, phenylalanine, acetoacetate, glutamate, glucose, and decreased histidine and citrate were associated with active metastasis. OPLS-DA yielded concordant metabolites. Subgroup analyses revealed distinct metabolic associations, e.g., for ICI therapy (citrate, RFE AUC 0.721) and BRAF status (acetate, RFE AUC 0.655), but limited performance for brain metastases (RFE AUC 0.553). ¹H-NMR serum metabolomics detects systemic metabolic alterations of active melanoma metastasis with moderate accuracy in a real-world setting. Identified disruptions in energy and amino acid metabolism offer pathobiological insights and warrant investigation for multimodal biomarker panels.

**Supplementary Information:**

The online version contains supplementary material available at 10.1038/s41598-026-37118-5.

## Introduction

The prognosis of melanoma critically depends on the presence of lymph node or distant metastases. 10-year survival rates drop from 85 to 95% for localized disease (American Joint Committee on Cancer [AJCC] Stage I/II)^1,2^ to 69% with lymphogenic metastasis (Stage III)^3^, while 1-year untreated survival with distant metastases ranges from 33 to 62%^4^. Early detection of new or progressive metastasis is crucial for timely therapy and improved survival^5,6^. However, current biomarkers predominantly reflect higher tumor burdens^7,8^, and are insufficient for detecting early metastasis. Similarly, imaging modalities such as Positron Emission Tomography (PET) scans have limitations for metastases < 5 mm^9^ and often lack specificity, making it difficult to distinguish metastases from benign inflammatory or infectious processes.

A hallmark of cancer is metabolic reprogramming, in which tumor cells adapt their metabolism (e.g., Warburg effect, altered amino acid, lipid, and nucleotide metabolism) to support rapid growth and metastasis^10,11^. These tumor-centric metabolic alterations also influence the systemic milieu, leading to measurable changes in patient body fluids^12^. Serum, an easily accessible biological sample, reflects these systemic changes and is therefore a promising medium for biomarker identification^12^. Serum metabolome analysis provides comprehensive insight into a patient’s metabolic status and can uncover signatures of cancer progression, including metastasis^13^.

Metabolomic profiling commonly utilizes ¹H-Proton Nuclear Magnetic Resonance (¹H-NMR) spectroscopy and Mass Spectrometry (MS), each with complementary strengths^14^. MS-based methods, particularly when coupled with chromatography (e.g., Liquid Chromatography-Tandem Mass Spectrometry [LC-MS/MS]), offer low detection limits for detecting metabolites^14,15^.

In contrast, ¹H-NMR spectroscopy provides highly reproducible, quantitative data with minimal sample preparation, making it a robust and practical platform for high-throughput analysis of clinical routine samples despite its poorer detection limits^16,17^. Consequently, this study utilized ¹H-NMR-based serum metabolomics to investigate systemic metabolic changes upon melanoma metastasis.

Studies on the melanoma metabolome have revealed significant metabolic alterations in tumor tissue, cell lines, and animal models^18,19^. Furthermore, pioneering work by Bayci et al.^12^ identified distinct serum profiles in advanced melanoma patients versus healthy controls using ¹H-NMR and MS, demonstrating proof-of-principle under controlled research conditions. However, translating such findings into real-world clinical practice, particularly for detecting active metastasis within a melanoma cohort rather than distinguishing from healthy controls, remains a critical gap. This translation is challenged by less standardized sample collection in routine care and the need for validation in larger, more practice-oriented patient cohorts. Addressing this, the present study investigates the applicability of ¹H-NMR-based serum metabolomics, focusing on soluble metabolites, to detect active metastasis in melanoma patients using a large cohort of samples collected during routine clinical care.

## Results

### Patient cohort and sample characteristics

The final cohort comprised 1698 serum samples from 963 unique melanoma patients (mean age 66.1 ± 14.6 years; 58.8% male). Tumor stage distribution (AJCC 8th Ed.) included: Stage 0 (0.1%), I (33.9%), II (16.0%), III (21.4%), and IV (25.5%) for cutaneous and mucosal melanoma; for the remaining 3.1% of patients (e.g., uveal or ciliary body melanoma), no AJCC staging was available. Active metastasis was present in 13.5% of samples (*n* = 229). A comparison of the baseline characteristics between samples with and without active metastasis is provided in Supplementary Table S5. Importantly, the two groups were well-matched regarding age and gender, minimizing potential demographic confounding. As expected, a significant difference was observed in the distribution of tumor stages between the groups (*p* < 0.001), reflecting the underlying disease progression.

These samples were split at the patient level into training (1021 samples, 578 patients) and test sets (677 samples, 385 patients), with no patient overlap. Baseline characteristics (Table 1) were generally well-balanced between the sets. Specifically, there were no significant differences in age, gender, the prevalence of active metastasis, systemic therapy status, or the distribution of metastasis locations. However, statistically significant differences were observed for the sample-level tumor stage distribution (*p* < 0.001) and the distribution of BRAF mutation status (*p* = 0.035).

**Table 1:** Baseline characteristics of the study cohort by training and test set allocation.

Characteristic	Training Set (*n* = 1021)	Test Set (*n* = 677)	*P*-Value
Age (years)	66.2 ± 14.7	65.9 ± 14.5	0.735
**Gender**			0.469
Female	428 (41.9%)	271 (40.0%)	
Male	593 (58.1%)	406 (60.0%)	
**Tumor Stage**			< 0.001
0	1 (0.1%)	0 (0.0%)	
IA	94 (9.2%)	50 (7.4%)	
IB	241 (23.6%)	191 (28.2%)	
IIA	68 (6.7%)	70 (10.3%)	
IIB	49 (4.8%)	38 (5.6%)	
IIC	22 (2.2%)	25 (3.7%)	
IIIA	44 (4.3%)	26 (3.8%)	
IIIB	93 (9.1%)	34 (5.0%)	
IIIC	78 (7.6%)	80 (11.8%)	
IIID	6 (0.6%)	2 (0.3%)	
IV	293 (28.7%)	140 (20.7%)	
Unknown/Not tested	32 (3.1%)	21 (3.1%)	
**BRAF Status**			0.035
Unknown/Not tested	512 (50.1%)	382 (56.4%)	
V600 mutated	246 (24.1%)	148 (21.9%)	
Wild-type	263 (25.8%)	147 (21.7%)	
**Systemic Therapy**			0.142
Immune Checkpoint Inhibitor (ICI)	114 (11.2%)	57 (8.4%)	
No systemic therapy	812 (79.5%)	566 (83.6%)	
Other Systemic Therapy	11 (1.1%)	9 (1.3%)	
Targeted Therapy	84 (8.2%)	45 (6.6%)	
**Active Metastasis**			0.451
No	889 (87.1%)	580 (85.7%)	
Yes	132 (12.9%)	97 (14.3%)	
**Brain Metastases (active)**			0.445
No	996 (97.6%)	665 (98.2%)	
Yes	25 (2.4%)	12 (1.8%)	
**Lymph Node Metastases (active)**			0.896
No	967 (94.7%)	643 (95.0%)	
Yes	54 (5.3%)	34 (5.0%)	
**Lung Metastases (active)**			0.759
No	990 (97.0%)	659 (97.3%)	
Yes	31 (3.0%)	18 (2.7%)	
**Liver Metastases (active)**			0.451
No	1003 (98.2%)	669 (98.8%)	
Yes	18 (1.8%)	8 (1.2%)	

Data are presented as Mean ± Standard Deviation (SD) for continuous variables and number (n) with percentage (%) for categorical variables. The training/test split was performed at the patient level. P-values were assessed using independent samples t-test for age and Chi-squared test for categorical variables. For clarity, metastasis locations are shown for the most frequent sites in the cohort.

### Identification of metabolite signatures for active metastasis

After preprocessing, 28 soluble ¹H-NMR quantified metabolites were available for feature selection from the training set (11 of 39 initial metabolites excluded for zero/near-zero variance; none for multicollinearity; a comprehensive list of all quantified analytes is provided in **Supplementary Table **S1).

Two multivariate approaches, Orthogonal Partial Least Squares Discriminant Analysis (OPLS-DA) and Recursive Feature Elimination (RFE) with Random Forest, were used for feature selection, followed by logistic regression. A scores plot visualizing the output of the OPLS-DA model on the training set is provided in **Supplementary Figure **S3. On the independent test set, the OPLS-DA-model achieved an Area Under the Curve (AUC) of 0.609 (95% CI: 0.550–0.668), corresponding to a sensitivity of 73.2% and a specificity of 42.6% at the optimal threshold. The RFE-model achieved an AUC of 0.630 (95% CI: 0.570–0.691), with a corresponding sensitivity of 35.1% and specificity of 85.2% for detecting active metastasis (Fig. 2). Training set AUCs were 0.664 and 0.707, respectively.

OPLS-DA identified 13 metabolites with a Variable Importance in Projection (VIP) score > 1 (top five: pyruvate, lactate, glucose, 3-hydroxybutyrate, acetone; Fig. 3A). RFE selected all 28 available metabolites as optimal (10-fold cross-validation indicated no performance improvement with fewer features); top five by Random Forest importance were pyruvate, glycine, glucose, alanine, and glutamate (Fig. 3B). Several top-ranked metabolites overlapped between the two feature selection methods (details in Supplemen﻿tary Table S2).

In the RFE-derived logistic regression model (28 metabolites), seven contributed significantly (*p* < 0.05, Wald test): pyruvate, phenylalanine, acetoacetate, glutamate, and glucose were associated with increased odds of active metastasis, while histidine and citrate were associated with decreased odds (Fig.4B, Supplementary Table S2). The OPLS-DA-model (13 metabolites) showed significant contributions from increased pyruvate, acetoacetate, and glutamate, and decreased histidine (Fig.4A, Supplementary Table S2). After Benjamini-Hochberg (BH) correction to control the False Discovery Rate (FDR), associations for pyruvate (both models) and histidine (OPLS-DA-model) remained robust (BH-FDR < 0.05; Supplementary Table S2). Descriptive statistics for key metabolites are provided in Supplementary Figure S1 and Supplementary Table S3.

### Subgroup analyses

Separate predictive models were developed for three predefined subgroups (Table 2; **Supplementary Table **S4**; Supplementary Figure **S2).

**Table 2:** Performance and key significantly differentiating metabolites of subgroup-specific predictive models.

Subgroup Analysis	Patient Selection Criteria (Samples)	Model Training/Testing (Samples (Unique Patients))	Modelling Approach	AUC on Test Set (95% CI)	Significant Metabolites (OR per SD, 95% CI, *p*-value)
ICI Therapy vs. Other Systemic Therapies	Samples from patients receiving systemic therapy (*N* = 320; ICI: *n* = 171, Other: *n* = 149)	Train: 187 (91 pat.), Test: 133 (60 pat.)	OPLS-DA based Logistic Regression	0.704 (0.608–0.799)	Citrate: OR 1.61 (1.10–2.35), *p* = 0.014; Isoleucine: OR 0.59 (0.35–0.97), *p* = 0.038
ICI Therapy vs. Other Systemic Therapies	Samples from patients receiving systemic therapy (*N* = 320; ICI: *n* = 171, Other: *n* = 149)	Train: 187 (91 pat.), Test: 133 (60 pat.)	RFE based Logistic Regression	0.721 (0.627–0.815)	Citrate: OR 1.55 (1.04–2.32), *p* = 0.031
Active Brain Met vs. Other Active Met Locations	Samples from patients with active metastatic disease (*N* = 229; Brain Met: *n* = 37, Other Met: *n* = 192)	Train: 131 (84 pat.), Test: 98 (55 pat.)	OPLS-DA based Logistic Regression	0.631 (0.507–0.754)	Acetone: OR 0.30 (0.09–1.00), *p* = 0.049
Active Brain Met vs. Other Active Met Locations	Samples from patients with active metastatic disease (*N* = 229; Brain Met: *n* = 37, Other Met: *n* = 192)	Train: 131 (84 pat.), Test: 98 (55 pat.)	RFE based Logistic Regression	0.553 (0.439–0.668)	Proline: OR 0.50 (0.25–0.98), *p* = 0.045*
BRAF Mutation vs. Other (in Active Met with Mutation Analysis)	Samples from patients with active metastasis and mutation analysis (*N* = 216; BRAF mutated: *n* = 107, Other: *n* = 109)	Train: 119 (78 pat.), Test: 97 (52 pat.)	OPLS-DA based Logistic Regression	0.599 (0.485–0.714)	Acetate: OR 4.95 (1.50–16.35), *p* = 0.009; Phenylalanine: OR 0.38 (0.17–0.84), *p* = 0.017
BRAF Mutation vs. Other (in Active Met with Mutation Analysis)	Samples from patients with active metastasis and mutation analysis (*N* = 216; BRAF mutated: *n* = 107, Other: *n* = 109)	Train: 119 (78 pat.), Test: 97 (52 pat.)	RFE based Logistic Regression	0.655 (0.545–0.765)	Acetate: OR 3.16 (1.18–8.49), *p* = 0.022*

For each subgroup analysis, separate logistic regression models based on features selected by Orthogonal Partial Least Squares Discriminant Analysis (OPLS-DA) or Recursive Feature Elimination (RFE) were trained and tested. Area Under the Curve (AUC) with 95% Confidence Interval (CI) is reported for the respective test sets. Statistically significant metabolites (*p* < 0.05, derived from Wald tests, with the 95% CI for the Odds Ratio (OR) per Standard Deviation (SD) not including 1.0) identified by these subgroup-specific logistic regression models are listed with their ORs, 95% CIs, and p-values. *Indicates statistical significance remaining after Benjamini-Hochberg correction for multiple testing within that specific sub-model (BH-FDR < 0.05). Abbreviations: AUC, Area Under the Curve; BH-FDR, Benjamini-Hochberg corrected False Discovery Rate; CI, Confidence Interval; ICI, Immune Checkpoint Inhibitor; Met, Metastases; OPLS-DA, Orthogonal Partial Least Squares Discriminant Analysis; OR, Odds Ratio; RFE, Recursive Feature Elimination; SD, Standard Deviation.

First, models differentiated immune checkpoint inhibitor (ICI) therapy (*n* = 171) from other systemic therapies (*n* = 149) among 320 samples from patients on systemic treatment. Performance was moderate (Test AUCs: OPLS-DA-model 0.704, RFE-model 0.721). Increased serum citrate was a consistent significant contributor (*p* < 0.05) in both models; decreased isoleucine also contributed significantly to the OPLS-DA-model (Table 2). No associations remained robust after BH-FDR correction (Supplementary Table S4).

Second, among 229 samples from patients with active metastases, models distinguished active brain metastases (*n* = 37) from other locations (*n* = 192) with limited to moderate performance (Test AUCs: OPLS-DA-model 0.631, RFE-model 0.553). The OPLS-DA-model identified decreased acetone, and the RFE-model decreased proline, as significant contributors (*p* < 0.05) (Table2). Only the proline association (RFE-model) remained robust after BH-FDR correction (BH-FDR = 0.045; Supplementary Table S4).

Third, for 216 samples from patients with active metastasis and known mutation status, models differentiated BRAF-mutated tumors (*n* = 107) from BRAF wild-type/other (*n* = 109) with limited to moderate ability (Test AUCs: RFE-model 0.655, OPLS-DA-model 0.599). Increased acetate was a significant contributor (*p* < 0.05) in both models, with decreased phenylalanine additionally significantly contributing to the OPLS-DA-model (Table 2). Only the acetate association (RFE-model) was robust after BH-FDR correction (BH-FDR = 0.044; Supplementary Table S4).

### Impact of Pre-analytical delays on the metastasis signature

To investigate the influence of pre-analytical sample handling, a sensitivity analysis was performed on subgroups stratified by pre-centrifugation time (≤ 120 min vs. >120min). The detailed results of these subgroup models are presented in Supplementary Tables S6 and S7.

This analysis yielded two key findings. First, in the ‘on-time’ subgroup of samples processed according to recommended SOPs (≤ 120 min), the predictive performance improved compared to the full cohort. The OPLS-DA model achieved an Area Under the Curve (AUC) of 0.67 (95% CI: 0.59–0.75), and the RFE model an AUC of 0.60 (95% CI: 0.51–0.69) on the validation data.

Second, in the ‘delayed’ subgroup (> 120 min), the model performance was reduced. The AUCs on the validation data were 0.55 (95% CI: 0.44–0.66) for the OPLS-DA model and 0.49 (95% CI: 0.38–0.59) for the RFE model, reflecting the impact of pre-analytical variability.

Despite the varying overall model performance, a core set of metabolic associations remained consistent. Our most robust finding, the positive association of pyruvate with active metastasis, was statistically significant across all subgroups and models. Furthermore, other key metabolites from our original signature, such as glutamate (positive association) and histidine (negative association), also showed consistent significant associations in most analytical scenarios. A detailed overview of all significant associations can be found in the Supplementary Tables (S6, S7).

## Discussion

This study demonstrated that ¹H-NMR-based serum metabolomics using routine clinical samples is capable of detecting active melanoma metastasis, albeit with only moderate discriminatory performance (test set AUCs: OPLS-DA-model 0.609, RFE-model 0.630). Furthermore, the divergent sensitivity and specificity profiles at the optimal thresholds likely reflect the flat ROC curves of these modest-performance models, indicating the absence of a single, robust classification cut-off. The models identified a metabolic signature for active metastasis, highlighted by robust associations (BH-FDR < 0.05) with increased pyruvate (both models) and decreased histidine (OPLS-DA-model). Other metabolites significantly (*p* < 0.05) contributing to this signature included increased odds for acetoacetate, glutamate, phenylalanine, and glucose, and decreased odds for citrate. These findings indicate systemic alterations in key metabolic pathways, particularly energy and amino acid metabolism, associated with active metastatic melanoma.

Our findings generally align with previous melanoma serum metabolomics studies^12,20–22^. Notably, our robust identification of decreased serum histidine in active metastasis is consistent with Bayci et al., who compared advanced melanoma patients to healthy controls using ¹H-NMR/MS under research conditions^12^.

It is important to distinguish the diagnostic goal of our study - identifying a signature of current active metastasis - from studies seeking prognostic signatures for future outcomes. For instance, the important work by Costantini et al. showed that pretreatment metabolite levels could predict overall survival in immunotherapy patients^23^. While our work serves as a foundational step to establish the feasibility of detecting metastatic signals in a heterogeneous real-world cohort, we fully agree that investigating the prognostic value of our findings for therapy response and survival is a critical future direction. Although our initial analysis highlighted lactate, a key metabolite in their study, it did not remain significant in our final model for metastatic activity.

Our more moderate model performance (test AUCs 0.609–0.630) compared to previous work (e.g., Bayci et al., AUC 0.82) is likely attributable to our more challenging intra-disease classification (active metastasis vs. no active metastasis) instead of a patient-versus-healthy control design^12^. Furthermore, while we observed increased acetoacetate and glutamate associated with active metastasis, Bayci et al. reported decreases. Such discrepancies likely arise from differing cohort definitions, our “real-world” ¹H-NMR approach on less standardized, non-fasting samples (focusing on soluble metabolites and excluding lipids), and potential variations in analytical methods. All these factors may contribute to the observed differences in metabolic profiles and AUCs compared to studies that include MS-based lipidomics^12,24^.

The identified metabolic signature indicates significant alterations in energy (glycolysis, Tricarboxylic Acid [TCA] cycle, Oxidative Phosphorylation [OXPHOS]) and amino acid metabolism with active melanoma metastasis. Notably, robustly elevated serum pyruvate suggests complex metabolic reprogramming beyond the classic Warburg effect (preferential glucose-to-lactate conversion)^25^. Although this result must be interpreted with caution due to potential pre-analytical glycolysis in non-stabilized tubes during sample processing delays, elevated systemic pyruvate may act as a critical metabolic node actively redirected in cancer. This re-direction can involve reduced mitochondrial pyruvate dehydrogenase (PDH) complex activity, potentially mediated by upregulation of pyruvate dehydrogenase kinase (PDK), thereby limiting TCA cycle influx and leading to pyruvate accumulation and release^26–28^. Accumulated pyruvate can also fuel anabolic pathways, such as transamination to alanine, thus linking glycolysis to amino acid metabolism and supporting the biosynthetic demands of proliferating cancer cells^10,11,29^.

Further evidence for reprogrammed energy metabolism includes increased odds for serum acetoacetate and glucose, and decreased odds for citrate. Elevated acetoacetate, a ketone body, may signify systemic shifts in fuel utilization, possibly reflecting increased fatty acid oxidation or ketogenesis^30,31^. Increased systemic glucose might indicate cancer-induced reprogramming of host glucose metabolism to ensure tumor supply, or direct promotion of cancer cell aggressiveness^32,33^. However, this finding warrants the same caution regarding pre-analytical glycolysis as mentioned above.

Decreased serum citrate, a key TCA cycle intermediate, suggests either TCA cycle dysfunction or, perhaps more compellingly, increased anaplerotic demand due to enhanced export for de novo fatty acid synthesis, often upregulated in cancer^10,34^. This export, driven by ATP-citrate lyase (ACLY), frequently overexpressed in cancers^35^, including melanoma^36^, supports the high lipogenic demands of metastatic cells, such as the need to build new cell membranes. This finding aligns with the established importance of lipid metabolism reprogramming in cancer progression^36,37^.

Increased serum glutamate may serve anaplerotic demands by fueling the TCA cycle via α-ketoglutarate, a key step in glutaminolysis often exploited by cancer cells^10,38,39^, or may reflect broader tumor-induced alterations in amino acid metabolism^10^.

Our findings also indicate dysregulated amino acid homeostasis. Decreased serum histidine, a robust observation, likely reflects increased tumor uptake and utilization^40,41^. In melanoma, L-type amino acid transporter 1 (LAT1) may facilitate histidine uptake. Once internalized, histidine can be used for protein synthesis or catabolized to support one-carbon metabolism, thereby contributing to nucleotide synthesis and cell proliferation and potentially depleting serum levels^42–44^.

In contrast, increased serum glutamate (potentially supporting tumor anaplerosis^38^, as discussed) and phenylalanine were observed. Elevated phenylalanine may result from impaired phenylalanine hydroxylase activity^45^ or increased systemic proteolysis in advanced cancer^46^. These patterns highlight the complex dysregulation of amino acid metabolism in cancer.

Collectively, these metabolic alterations in serum point to a multifaceted dysregulation of central carbon metabolism, mitochondrial function, and amino acid homeostasis in metastatic melanoma.

To investigate potential metabolic distinctions in specific clinical scenarios, exploratory subgroup analyses were conducted. These analyses, aimed at differentiating patients based on ICI therapy, brain metastasis presence, or BRAF mutation status, generally showed limited to moderate predictive performances (Table 2).

For differentiating patients on immune checkpoint inhibitor (ICI) therapy (Test AUCs ~ 0.7–0.72), increased serum citrate was a consistent (though not BH-FDR robust) finding. This contrasts with decreased citrate in the main active metastasis model. A plausible explanation involves the metabolic reprogramming essential for activated T-cells under ICI therapy. These cells undergo significant metabolic shifts to support proliferation and effector functions, including increased demand for citrate as a substrate for de novo fatty acid synthesis (e.g., for membrane biogenesis)^47^. Thus, elevated systemic citrate might indirectly reflect robust immune cell engagement rather than a direct tumor metabolic output. Decreased isoleucine (OPLS-DA-model) was less robust.

Models distinguishing active brain metastases (*n* = 37) performed poorly (Test AUCs ~ 0.55–0.63). Although decreased proline (RFE-model) remained robust after FDR correction, the clinical relevance of this finding is limited by the low overall AUC and small subgroup size. The observed association between decreased acetone (OPLS-DA-model) and potential ketone body uptake by brain metastases remains speculative given confounding factors and the influence of the blood-brain barrier^48,49^.

Differentiating BRAF-mutated tumors in active metastasis (Test AUCs ~ 0.60–0.66) identified increased serum acetate (RFE-model). While this may relate to metabolic adaptations in BRAF-mutant melanoma, such as increased reliance on OXPHOS^50^, the mechanistic link remains speculative. Elevated serum acetate could reflect altered systemic metabolism or increased tumor utilization of acetate, potentially involving acetyl-CoA synthetase 2 (ACSS2). ACSS2, which converts acetate to acetyl-CoA, fueling the TCA cycle and OXPHOS^51,52^. Decreased phenylalanine (OPLS-DA-model) was less clear.

Overall, these subgroup analyses highlight challenges in identifying robust serum biomarkers for specific subclassifications, although some findings warrant further exploration.

While this study demonstrates the feasibility of detecting systemic metabolic alterations of active melanoma metastasis via ¹H-NMR in a real-world setting, the primary models (AUCs 0.609–0.630) are not sufficiently robust for standalone diagnostic use. Nevertheless, the identified metabolic changes offer clinical and research implications.

The metabolic perturbations in energy and amino acid pathways, particularly robust findings like increased pyruvate and decreased histidine, alongside other contributors (phenylalanine, acetoacetate, glutamate, glucose, citrate), deepen our understanding of host-tumor interactions and may unveil therapeutic targets^53^. Further research is needed to elucidate the underlying mechanisms.

The identified metabolic signature or its components could contribute to multimodal biomarker panels, potentially improving risk stratification or disease detection when combined with clinical, imaging, and other omics data^28,54^. For instance, associations of citrate with ICI therapy or acetate with BRAF mutations warrant investigation for patient stratification or therapy monitoring.

Future research should prioritize prospective, multicenter validation in larger, stringently characterized cohorts. A key priority for such a prospective study will be to establish a defined ‘time zero’ for all patients, allowing for a methodologically sound investigation of the prognostic value of these metabolic signatures for key clinical outcomes, including overall survival and therapy response. Furthermore, MS-based lipidomics^36,37^, exploring the interplay with NMR-derived systemic inflammation markers such as GlycA, performing longitudinal sampling for dynamic biomarkers, exploring other biofluids (e.g., urine; CSF for brain metastases), and more granular analysis of treatment impacts are also warranted.

Notable strengths of the study include the large single-center melanoma cohort (1698 samples, 963 unique patients) for ¹H-NMR serum metabolomics targeting active metastasis, providing a robust foundation for model development. Importantly, patient-level training/test splitting ensured independent validation and a rigorous assessment of model performance. The “real-world” sample collection during routine care enhances generalizability of robust findings, despite inherent heterogeneity. Furthermore, the pragmatic ¹H-NMR approach on serum, focusing on soluble metabolites to mitigate non-fasting lipid variability makes this method well-suited for larger-scale applications. The concordance between the OPLS-DA and RFE-based models for key metabolites strengthens confidence in these alterations. Both methods identified increased odds for pyruvate, acetoacetate, and glutamate, and decreased odds for histidine. Exploratory subgroup models also provided initial insights into metabolic heterogeneity.

Several limitations warrant consideration. The study’s primary constraints arise from its single-center, “real-world” design. This led to a tumor stage imbalance between the training and test sets and resulted in imbalanced group sizes for the primary analysis (1469 vs. 229 samples), a characteristic that reflects the clinical prevalence of active disease in a follow-up cohort. This design also introduced broad pre-analytical variability from non-standardized collection (e.g., fasting status, processing times); both factors may have contributed to the moderate model performance. While serum generally remains a robust matrix for broad metabolomic profiling even with processing delays^55^, our dedicated sensitivity analysis quantitatively confirmed the specific impact on our metastasis signature. This analysis demonstrated that model performance was reduced in the subgroup with prolonged pre-centrifugation delays (> 120 min; AUCs down to 0.49), underscoring the critical importance of strict SOPs for detecting this particular signal. Concurrently, the analysis also validated our primary findings, as model performance improved in the SOP-compliant subgroup (≤ 120 min; AUC up to 0.67), and the core signature around pyruvate remained robust. Further limitations include the lower analytical sensitivity of ¹H-NMR compared to MS and our focus on soluble metabolites; limited statistical power in small subgroups; a high multiple testing burden due to the number of features tested; reliance on clinical definitions for metastasis; and the cross-sectional design, which precludes causal inference.

In conclusion, this study demonstrates that ¹H-NMR-based serum metabolomics can detect systemic metabolic alterations of active metastasis in a large, ‘real-world’ melanoma cohort. While the moderate discriminatory performance highlights the challenges of clinical and pre-analytical variability and precludes standalone diagnostic use, our findings offer important insights. The identified signature of dysregulated energy and amino acid metabolism provides a strong rationale for future research. The primary clinical utility of these findings will likely be realized through the integration of key metabolites, such as pyruvate and histidine, into multimodal biomarker panels alongside established clinical and imaging data. Future research should therefore focus on the prospective validation of such multimodal approaches and the investigation of this signature’s potential for monitoring therapy response. Our work underscores both the potential and the current limitations of serum metabolomics in routine clinical practice, paving the way for more refined and integrated biomarker strategies in melanoma.

### Methods

#### Ethics statement

The study protocol was approved by the Ethics Committee at the Technische Universität Dresden (Approval No. BO-EK-265062022). The study was conducted in accordance with the Declaration of Helsinki, and participating patients provided written informed consent for the use of their biomaterials for research purposes. The study was registered at clinicaltrials.gov (Identifier: NCT06765850).

### Study design, cohort, and data collection

**Fig. 1:**
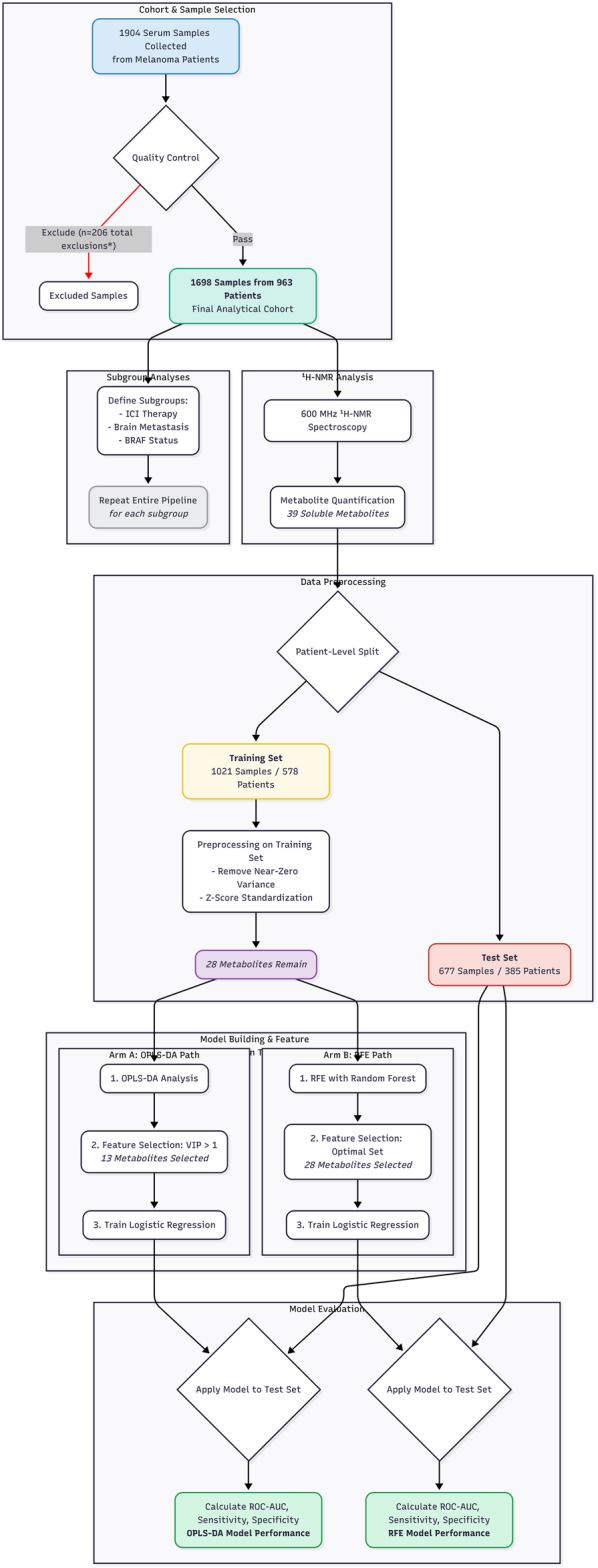
Study Flowchart.The diagram summarizes the workflow of the study, from patient cohort definition and sample exclusion to data preprocessing, patient-level splitting into training and test sets, and the parallel analytical pipelines for model building (OPLS-DA and RFE). The final evaluation on the independent test set and the separate subgroup analyses are also depicted. *Note: The 206 total exclusions represent partly overlapping criteria.

An overview of the study design, patient flow, and analytical workflow is provided in Fig. 1. This single-center prospective observational study included adult melanoma patients (cutaneous, mucosal, or uveal) aged ≥ 18 years from a Skin Cancer Center associated with a German university hospital, enrolled between July 2022 and December 2023. Patients were under routine follow-up (Stage IB+), receiving systemic therapy for metastatic disease, or undergoing surgery. Serum samples and associated data were prospectively collected for this research project.

The primary endpoint, “Active Metastasis,” was defined as new or progressive metastases identified through a retrospective review of interdisciplinary Tumor Board decisions and clinical history within a three-month window surrounding each sample collection. This study employed a cross-sectional design, treating each of the 1698 samples as an independent metabolic ‘snapshot’. Samples were collected during routine clinical appointments, independent of specific events like initial diagnosis or therapy initiation, with a minimum interval of three months between repeated samples from the same patient. This ‘real-world’ approach was chosen to capture the metabolic state of a heterogeneous patient cohort under routine care. Subgroup analyses explored differences based on ICI therapy, brain metastases, and BRAF status.

The final analytical cohort comprised 1698 serum samples from 963 unique patients (552 patients with one sample, 225 with two, 100 with three, 46 with four, 28 with five, and 12 with six).

### Sample collection and handling

Serum samples were collected during routine clinical practice. Blood was drawn into 7.5 ml serum tubes. Collection was non-standardized regarding time of day or fasting status, reflecting real-world conditions. Collected serum tubes were transported to the local, ethically-approved Biobank, where serum was prepared according to established standard operating procedures for metabolomics^56,57^ (clotting, centrifugation at 2000 g, room temperature, 10 min, no break). Serum aliquots were stored in liquid nitrogen vapor (−196 °C) until ¹H-NMR analysis.

**Fig. 2:**
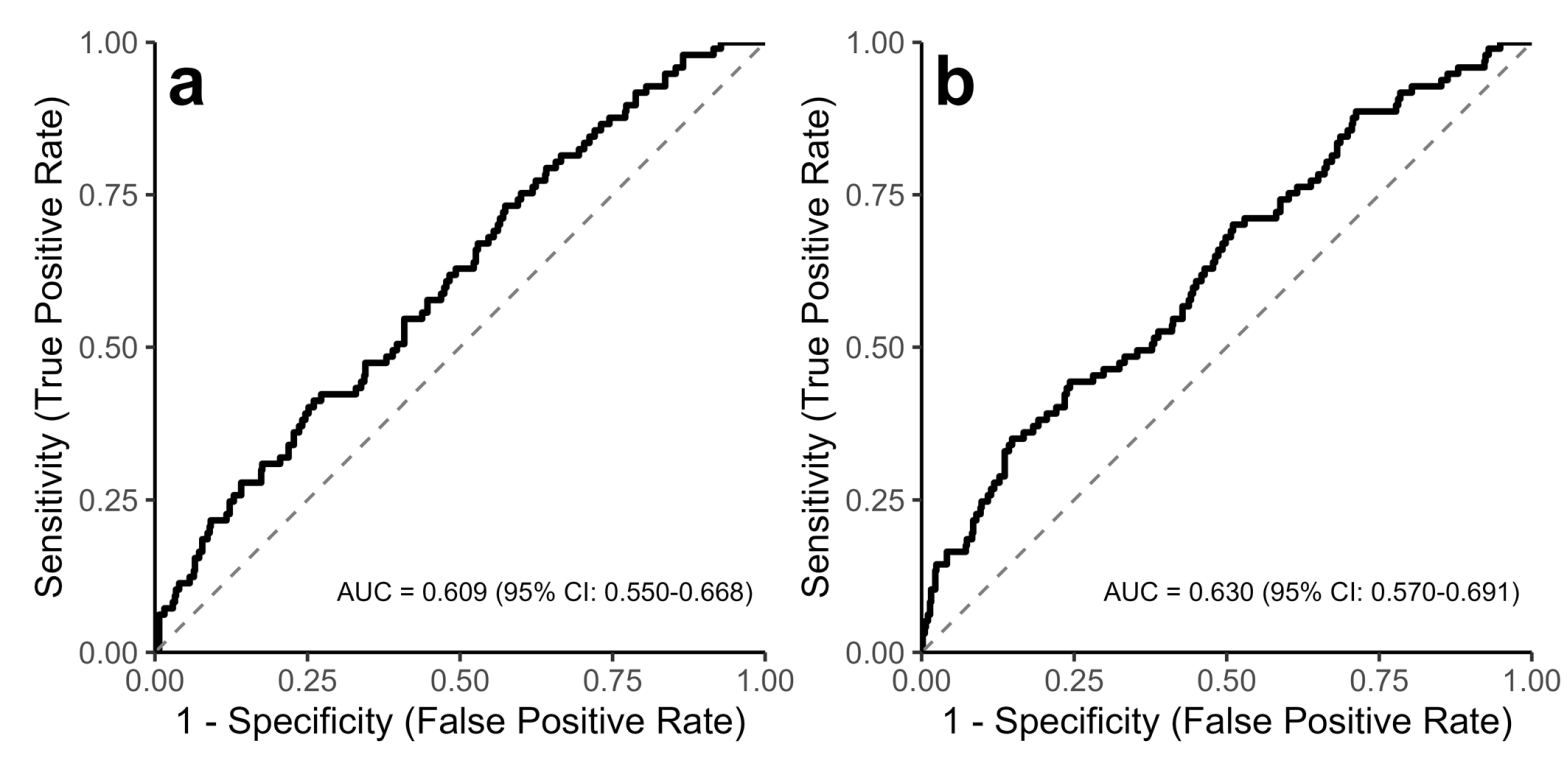
Receiver Operating Characteristic (ROC) curves for models discriminating active metastasis on the independent test set. **(a)** Performance of the logistic regression model based on Orthogonal Partial Least Squares Discriminant Analysis (OPLS-DA) feature selection (AUC = 0.609, 95% CI: 0.550–0.668; solid black line). **(b)** Performance of the logistic regression model based on Recursive Feature Elimination (RFE) with Random Forest feature selection (AUC = 0.630, 95% CI: 0.570–0.691; solid black line). For both panels, the diagonal grey dashed line represents the performance of a random classifier (AUC = 0.5). AUC, Area Under the Curve; CI, Confidence Interval.

**Fig. 3:**
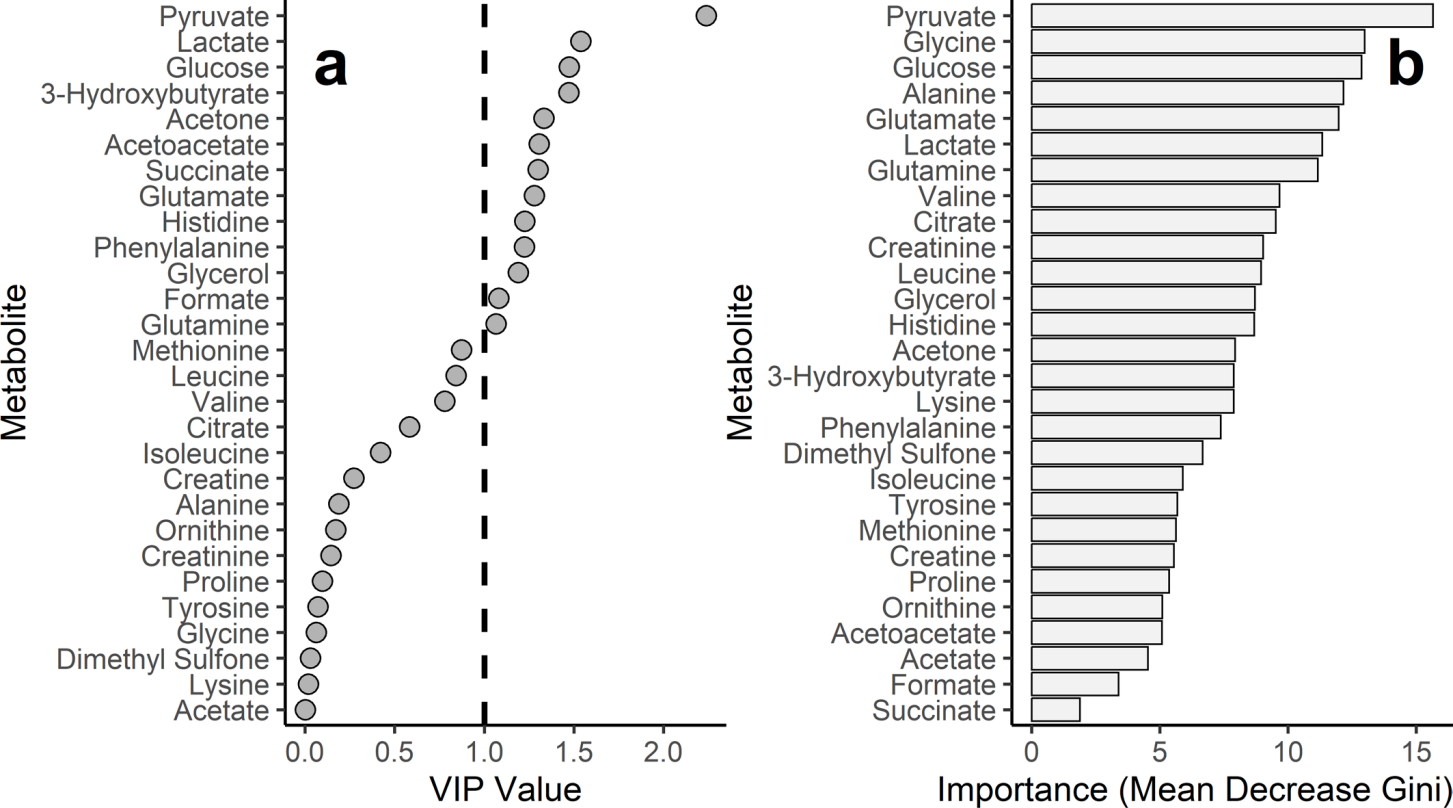
Feature importance metrics for 28 soluble metabolites in discriminating active metastasis status, derived from models trained on the training set. **(a)** Variable Importance in Projection (VIP) scores from Orthogonal Partial Least Squares Discriminant Analysis (OPLS-DA). Metabolites exceeding the standard threshold for feature importance (VIP > 1) are indicated. The dashed horizontal line represents this threshold. **(b)** Random Forest Feature Importance (Mean Decrease Gini) for the 28 metabolites selected by Recursive Feature Elimination (RFE). Metabolites are ordered by decreasing importance from top to bottom.

**Fig. 4:**
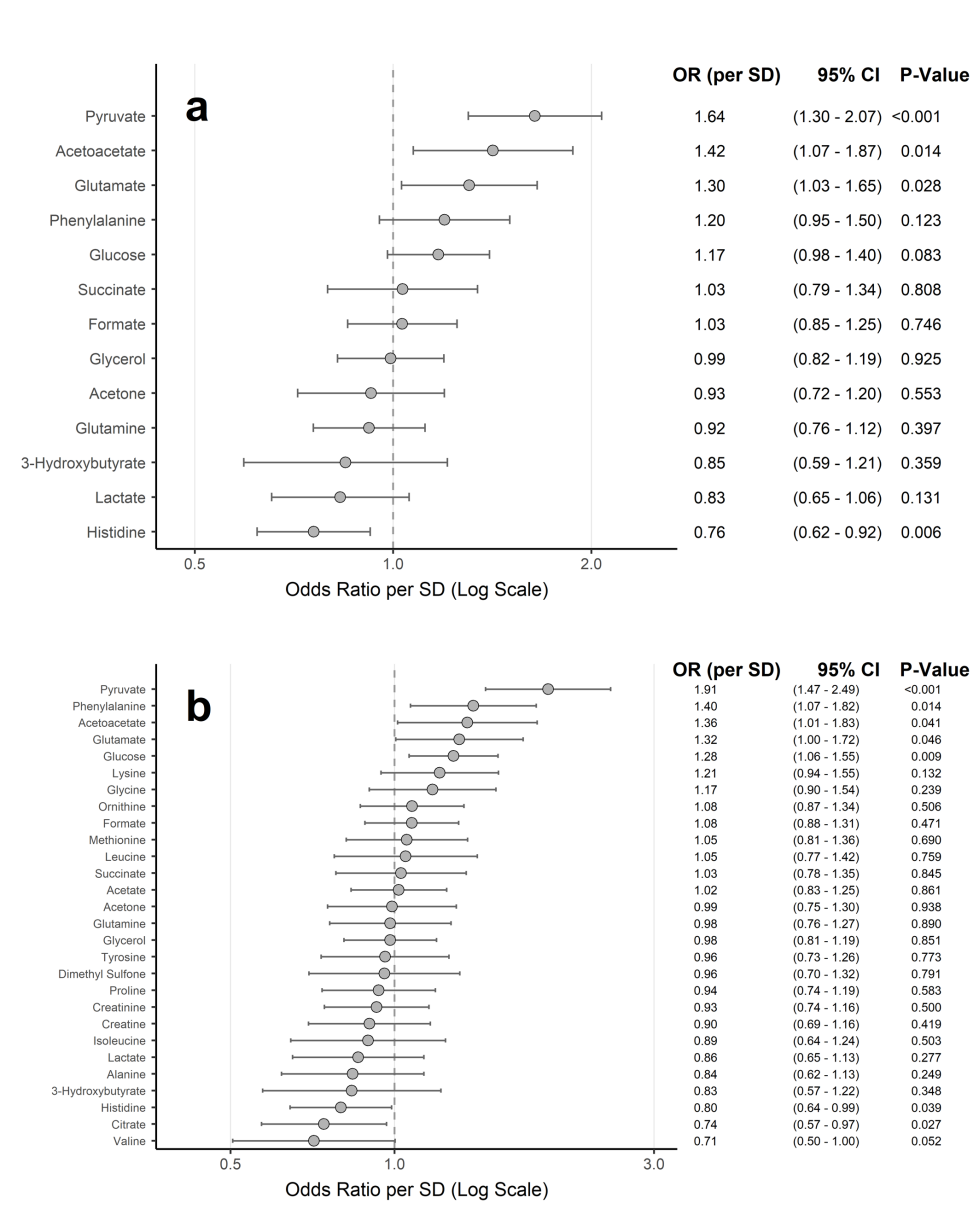
Metabolite associations with active metastasis status identified by logistic regression models based on OPLS-DA and RFE feature selection. Forest plots display Odds Ratios (ORs) with 95% Confidence Intervals (CIs) per one standard deviation (SD) increase in metabolite concentration for predicting active metastasis. Circles represent the ORs, and horizontal lines indicate the 95% CIs. Metabolites are sorted by their OR per SD (descending). The vertical dashed line at OR = 1 indicates no association. Numerical values for ORs, 95% CIs, and P-values are presented to the right of each plot within the figure panels. **(a)** Associations for the thirteen soluble metabolites selected by Orthogonal Partial Least Squares Discriminant Analysis (OPLS-DA) (Variable Importance in Projection [VIP] > 1). **(b)** Associations for the 28 soluble metabolites included in the final Recursive Feature Elimination (RFE) selected model. OR, Odds Ratio; CI, Confidence Interval; SD, Standard Deviation; OPLS-DA, Orthogonal Partial Least Squares Discriminant Analysis; VIP, Variable Importance in Projection; RFE, Recursive Feature Elimination.

### ¹H-NMR analysis: spectroscopy, processing, and quantification

Sample preparation and ¹H-NMR spectroscopy were performed according to established protocols^56–59^ and Bruker In Vitro Diagnostics research (IVDr) Methods protocols. Briefly, serum samples were prepared with a buffer containing 2.3 mM Trimethylsilylpropansulfonate (TMSP) as an internal standard. Measurements were performed on a Bruker BioSpin 600 MHz AVANCE NEO (BBI-Probe, SampleJet at 4 °C). Spectra were acquired at 310 K using pulse sequences optimized for metabolic profiling, including 1D Carr-Purcell-Meiboom-Gill (CPMG; cpmgpr1d) and 1D Nuclear Overhauser Effect Spectroscopy (NOESY-presat; noesygppr1d), with quantitative calibration per Bruker IVDr.

Raw spectra were processed (Fourier transformation, phasing, baseline correction, referencing to TMSP at 0.0 ppm) using Bruker TopSpin 3.2. Metabolite quantification was performed using Bruker’s B.I.Quant-PS (v2.0.0; 39 soluble parameters) and B.I.LISA (v1.0.0; 111 lipid subclass parameters) panels, based on spectral deconvolution against reference libraries. Although lipoprotein data were quantified, they were deliberately excluded from all subsequent analyses. This decision was made because samples were collected in a non-fasting state during routine clinical care, which is known to introduce significant variability in lipid and lipoprotein profiles, potentially obscuring the more subtle metabolic signals related to cancer progression. Therefore, this study focused exclusively on the analysis of soluble metabolites.

### Data preprocessing and quality control

From 1904 initial serum measurements, quality checks excluded 4 non-melanocytic tumor samples and 3 with incomplete metabolite data, yielding 1897 complete melanoma samples. Further exclusions (time to first centrifugation ≥ 300 min, *n* = 89; hemolytic/lipemic serum, *n* = 13; active second tumor, *n* = 106; criteria partly overlapping) yielded 1698 samples from 963 unique patients for analysis. Mean time to centrifugation for these samples was 131.9 ± 72.7 min.

Although some patients contributed multiple samples over time, the sparse and irregular nature of this longitudinal data was insufficient for a formal time-series analysis. Therefore, to appropriately handle the statistical non-independence of repeated measures from the same individual, we treated each sample as a cross-sectional snapshot and implemented a strict patient-level split (random seed: 1234). The cohort was divided into a training (1021 samples, 578 patients) and a test set (677 samples, 385 patients), ensuring no patient overlap between the sets. This approach ensures that the model is evaluated on samples from patients entirely unseen during training, providing a robust estimate of its generalizability. In the training set, variables with zero/near-zero variance (R function nearZeroVar, default settings) were excluded. Metabolite data were Z-score normalized using training set parameters, then applied to the test set. For multicollinearity, one variable from pairs with Pearson correlation > 0.9 in the training set was removed from both sets.

### Statistical analysis and model Building

Analyses used R (version 4.4.3; R Core Team, 2025; key packages: caret for RFE, ropls for OPLS-DA, randomForest, pROC). Baseline characteristics (age, sex, tumor stage, active metastasis) were compared between training/test sets (t-tests/Chi-squared tests; *p* < 0.05 for significance).

On the training set, two feature selection approaches were used: OPLS-DA (VIP > 1 selection criterion) and RFE with Random Forest (10-fold cross-validation, rfFuncs metric) were employed to identify optimal metabolite subsets. Subsequently, logistic regression models were trained using OPLS-DA- or RFE-selected metabolites. Those predicting active metastasis in the overall cohort constituted the ‘main models’. Performance was evaluated on the independent test set (ROC analysis, AUC). Additionally, sensitivity and specificity were determined at the optimal threshold defined by the Youden index.

For metabolites included in the final logistic regression models, Odds Ratios (ORs) per standard deviation (SD) increase (95% CIs) were calculated. Statistical significance of each metabolite’s contribution was assessed using the Wald test (*p* < 0.05). These p-values were subsequently adjusted for multiple comparisons using the BH procedure to control the FDR, with associations considered robust at BH-FDR < 0.05.

For predefined patient subgroups (ICI therapy vs. other systemic treatments; active brain vs. other active metastases; BRAF-mutated vs. BRAF wild-type/other in active metastasis with mutation status), distinct OPLS-DA and RFE-based logistic regression models were independently developed using the respective training set subgroups. The models were then evaluated on the corresponding test set subsets, mirroring the procedure of the main model, including BH-FDR correction.

### Sensitivity analysis for pre-analytical variability

To address the potential impact of pre-analytical sample handling delays, we performed a sensitivity analysis based on the pre-centrifugation time. We stratified our entire cohort into two subgroups: an ‘on-time’ group (processing time ≤ 120 minutes), reflecting adherence to strict SOPs, and a ‘delayed’ group (processing time > 120 and < 300 min). For both subgroups, we independently repeated the entire analysis workflow, including feature selection (OPLS-DA and RFE) and the development of logistic regression models. The performance of these subgroup models was assessed on their respective validation sets.

## Supplementary Information

Below is the link to the electronic supplementary material.

Supplementary Material 1

## Data Availability

The reduced dataset, comprising quantified ¹H-NMR serum metabolite concentrations and associated de-identified clinical metadata necessary to reproduce the main findings of this study, is publicly available in a Zenodo repository at https://doi.org/10.5281/zenodo.15676686 (DOI: 10.5281/zenodo.15676686). The R scripts used for data preprocessing and statistical analysis are publicly available on GitHub at https://github.com/ffgellrich/Melanoma-Metabolomics-R-Scripts.In accordance with FAIR data principles, the full raw ¹H-NMR spectral data from the NOESY experiments, on which the analysis is based, have also been deposited in the aforementioned Zenodo repository. The raw data are provided as compressed archives containing the complete Bruker experiment directories (including FID, acquisition, and processing parameter files).Further derived data supporting the findings of this study are available within the article and its Supplementary Information files. This study was also registered at clinicaltrials.gov (Identifier: NCT06765850).

## Abbreviations

¹H-NMR: Proton nuclear magnetic resonance
AJCC: American joint committee on cancer
AUC: Area under the curve
BH-FDR: Benjamini-hochberg false discovery rate
CPMG: Carr-purcell-meiboom-gill
ICI: Immune checkpoint inhibitor
IVDr: In Vitro Diagnostics research (Bruker platform)
LC-MS/MS: Liquid Chromatography-Tandem Mass Spectrometry
MS: Mass Spectrometry
NOESY: Nuclear overhauser effect spectroscopy
OPLS-DA: Orthogonal partial least squares discriminant analysis
OXPHOS: Oxidative Phosphorylation
PDH: Pyruvate Dehydrogenase
PDK: Pyruvate Dehydrogenase Kinase
PET: Positron emission tomography
RFE: Recursive feature elimination
ROC: Receiver operating characteristic
TCA: Tricarboxylic acid (Cycle)
TMSP: Trimethylsilylpropansulfonate
VIP: Variable importance in projection
