# Correction: ^1^H-NMR serum metabolomic profiling from clinical routine identifies signatures of progressive melanoma metastasis

**DOI:** 10.1038/s41598-026-57962-9

**Published:** 2026-07-08

**Authors:** Frank Friedrich Gellrich, Cosima Hufnagel, Alexander M. Funk, Sophie Jonas, Heidi Altmann, Sarah Hobelsberger, Julian Steininger, Ricarda Rauschenberg, Marlene Garzarolli, Sophia Lehr, Lea Pöschman, Paula Biedermann, Julia Sophie Schatz, Christina Feige, Alpaslan Tasdogan, Triantafyllos Chavakis, Stefan Beissert, Friedegund Meier, Peter Mirtschink, Gerald Steiner

**Affiliations:** 1 Department of Dermatology, Faculty of Medicine, University Hospital Carl Gustav Carus, Technische Universität Dresden, Dresden, Germany; 2 Skin Cancer Center at the University Cancer Center, National Center for Tumor Diseases, Dresden, Germany; 3 BioBank Dresden, Faculty of Medicine, University Hospital Carl Gustav Carus, Technische Universität Dresden, Dresden, Germany; 4 National Center for Tumor Diseases, Dresden (NCT/UCC), Dresden, Germany; 5 Department of Anesthesia and Intensive Care, Clinical Sensoring and Monitoring, Faculty of Medicine Carl Gustav Carus, University Hospital, Technische Universität Dresden, Dresden, Germany; 6 Institute for Clinical Chemistry and Laboratory Medicine, Technische Universität Dresden, 01307 Dresden, Germany; 7 Department of Dermatology, University Hospital Essen and German Cancer Consortium (DKTK), Partner Site Essen, Essen, Germany; 8 National Center for Tumour Diseases (NCT-West), Campus Essen & Research Alliance Ruhr, Research Center One Health, University Duisburg-Essen, Essen, Germany

Correction to: *Scientific Reports* 10.1038/s41598-026-37118-5, published online 30 January 2026

In the original version of this Article, authors Ricarda Rauschenberg, Marlene Garzarolli, Sophia Lehr, Lea Pöschman, Paula Biedermann, and Julia Sophie Schatz were omitted from the author list. The author list has now been corrected.

