# Supplementary material for: ¹H-NMR serum metabolomic profiling from clinical routine identifies signatures of progressive melanoma metastasis
Source: Sci Rep. 2026 Jan 30;16:5263. doi: 10.1038/s41598-026-37118-5 (PMC12881348; doi:10.1038/s41598-026-37118-5)
Supplement: Supplementary file 1 — Supplementary Material 1 [file 41598_2026_37118_MOESM1_ESM.docx]

# Title Page

## Title

Supplementary Information for: ¹H-NMR Serum Metabolomic Profiling from Clinical Routine Identifies Signatures of Progressive Melanoma Metastasis

## Autoren

Frank Friedrich Gellrich^1,2*^, 0000-0002-2164-4644, frankfriedrich.gellrich@ukdd.de

Cosima Hufnagel^1,2^, 0009-0001-0859-2915, cosima.hufnagel@mailbox.tu-dresden.de

Alexander M. Funk^6^, 0000-0002-7248-4599, alexander.funk@ukdd.de

Sophie Jonas^6^, ORCID not available, sophie.jonas@ukdd.de

Heidi Altmann^3,4^, 0000-0003-1967-0330, Heidi.Altmann@ukdd.de

Sarah Hobelsberger^1,2^, 0000-0001-5703-324X, Sarah.Hobelsberger@ukdd.de

Julian Steininger^1,2^, 0000-0001-9094-4512, Julian.Steininger@ukdd.de

Ricarda Rauschenberg^1,2^, [0009-0001-4054-4024](https://orcid.org/0009-0001-4054-4024), ricarda.rauschenberg@ukdd.de

Marlene Garzarolli^1,2^, ORCID not available, marlene.garzarolli@ukdd.de

Sophia Lehr^1,2^, [0000-0002-2639-6010](https://orcid.org/0000-0002-2639-6010), sophia.lehr@ukdd.de

Lea Pöschman^1,2^, 0009-0007-4126-8248, lea.poeschmann@ukdd.de

Paula Biedermann^1,2^, 0000-0002-0576-0565, paula.biedermann@ukdd.de

Julia Sophie Schatz^1,2^, ORCID not available, Julia.Schatz@ukdd.de

Christina Feige^1,2^, ORCID not available, Christina.Feige@ukdd.de

Alpaslan Tasdogan^7,8^, 0000-0003-2543-852X, Alpaslan.Tasdogan@uk-essen.de

Triantafyllos Chavakis^6^, 0000-0002-1869-5141, triantafyllos.chavakis@ukdd.de

Stefan Beissert^1^, 0000-0002-8327-8475, Stefan.Beissert@ukdd.de

Friedegund Meier^1,2^, 0000-0003-4340-9706, Friedegund.Meier@ukdd.de

Peter Mirtschink^6,†^, 0000-0001-5832-1673, Peter.Mirtschink@ukdd.de

Gerald Steiner^5,†^, 0000-0002-7625-343X, gerald.steiner@tu-dresden.de

^*^Corresponding author

^†^Contributed equally

^1^Department of Dermatology, University Hospital Carl Gustav Carus, Technische Universität Dresden, Dresden, Germany

^2^Skin Cancer Center at the University Cancer Center and National Center for Tumor Diseases Dresden, Germany

^3^BioBank Dresden, Faculty of Medicine and University Hospital Carl Gustav Carus, Technische Universität Dresden, Dresden, Germany

^4^National Center for Tumor Diseases, Dresden (NCT/UCC), Dresden, Germany

^5^Department of Anesthesia and Intensive Care, Clinical Sensoring and Monitoring, Faculty of Medicine Carl Gustav Carus, University Hospital, Technische Universität Dresden, Dresden, Germany

^6^Institute for Clinical Chemistry and Laboratory Medicine, Technische Universität Dresden, Dresden 01307, Germany

^7^Department of Dermatology, University Hospital Essen & German Cancer Consortium (DKTK), Partner Site Essen, Essen, Germany

^8^National Center for Tumour diseases (NCT-West), Campus Essen & Research Alliance Ruhr, Research Center One Health, University Duisburg-Essen, Essen, Germany

## Corresponding author

Frank Friedrich Gellrich

# Supplementary

## Supplementary Table S1

Supplementary Table S1: Comprehensive list of quantified serum metabolites and lipoprotein parameters.

| **Main Category** | **Subcategory** | **Metabolite / Parameter** |
| --- | --- | --- |
| Soluble Metabolites | Amino Acids and Related Compounds | Alanine |
|  |  | Asparagine |
|  |  | Creatine |
|  |  | Creatinine |
|  |  | Glutamate |
|  |  | Glutamine |
|  |  | Glycine |
|  |  | Histidine |
|  |  | Isoleucine |
|  |  | Leucine |
|  |  | Lysine |
|  |  | Methionine |
|  |  | N,N-Dimethylglycine |
|  |  | Ornithine |
|  |  | Phenylalanine |
|  |  | Proline |
|  |  | Sarcosine |
|  |  | Threonine |
|  |  | Tyrosine |
|  |  | Valine |
|  |  | 2-Aminobutyrate |
|  |  | 2-Hydroxybutyrate |
|  | Organic Acids, TCA Cycle Intermediates, and Glycolysis Products | Acetate |
|  |  | Citrate |
|  |  | Formate |
|  |  | Lactate |
|  |  | Pyruvate |
|  |  | Succinate |
|  |  | 2-Oxoglutarate |
|  | Ketone Bodies | 3-Hydroxybutyrate |
|  |  | Acetoacetate |
|  |  | Acetone |
|  | Sugars and Sugar Alcohols | Galactose |
|  |  | Glucose |
|  |  | Glycerol |
|  | Choline and Related Compounds | Choline |
|  |  | Trimethylamine N-oxide (TMAO) |
|  | Other Soluble Metabolites | Dimethyl Sulfone |
|  |  | Ethanol |
| Lipids and Lipoproteins | Main Clinical Lipid Parameters | Total Triglycerides (TG) |
|  |  | Total Cholesterol (Chol) |
|  |  | LDL Cholesterol (LDL-Chol) |
|  |  | HDL Cholesterol (HDL-Chol) |
|  |  | Apolipoprotein A1 (Apo-A1) |
|  |  | Apolipoprotein A2 (Apo-A2) |
|  |  | Apolipoprotein B100 (Apo-B100) |
|  |  | LDL Cholesterol / HDL Cholesterol Ratio |
|  | Lipoprotein Particle Concentrations | Total ApoB-containing Particles |
|  |  | VLDL Particles |
|  |  | IDL Particles |
|  |  | LDL Particles (Total) |
|  | Lipoprotein Particle Concentrations (LDL Subfractions) | LDL-1 Particles |
|  |  | LDL-2 Particles |
|  |  | LDL-3 Particles |
|  |  | LDL-4 Particles |
|  |  | LDL-5 Particles |
|  |  | LDL-6 Particles |
|  | Lipid Concentrations in VLDL | Triglycerides in VLDL |
|  |  | Cholesterol in VLDL |
|  |  | Free Cholesterol in VLDL |
|  |  | Phospholipids in VLDL |
|  |  | Apolipoprotein B in VLDL |
|  | Lipid Concentrations in VLDL Subfractions | Triglycerides in VLDL-1 |
|  |  | Triglycerides in VLDL-2 |
|  |  | Triglycerides in VLDL-3 |
|  |  | Triglycerides in VLDL-4 |
|  |  | Triglycerides in VLDL-5 |
|  |  | Cholesterol in VLDL-1 |
|  |  | Cholesterol in VLDL-2 |
|  |  | Cholesterol in VLDL-3 |
|  |  | Cholesterol in VLDL-4 |
|  |  | Cholesterol in VLDL-5 |
|  |  | Free Cholesterol in VLDL-1 |
|  |  | Free Cholesterol in VLDL-2 |
|  |  | Free Cholesterol in VLDL-3 |
|  |  | Free Cholesterol in VLDL-4 |
|  |  | Free Cholesterol in VLDL-5 |
|  |  | Phospholipids in VLDL-1 |
|  |  | Phospholipids in VLDL-2 |
|  |  | Phospholipids in VLDL-3 |
|  |  | Phospholipids in VLDL-4 |
|  |  | Phospholipids in VLDL-5 |
|  | Lipid Concentrations in IDL | Triglycerides in IDL |
|  |  | Cholesterol in IDL |
|  |  | Free Cholesterol in IDL |
|  |  | Phospholipids in IDL |
|  |  | Apolipoprotein B in IDL |
|  | Lipid Concentrations in LDL | Triglycerides in LDL |
|  |  | Free Cholesterol in LDL |
|  |  | Phospholipids in LDL |
|  |  | Apolipoprotein B in LDL |
|  | Lipid Concentrations in LDL Subfractions | Triglycerides in LDL-1 |
|  |  | Triglycerides in LDL-2 |
|  |  | Triglycerides in LDL-3 |
|  |  | Triglycerides in LDL-4 |
|  |  | Triglycerides in LDL-5 |
|  |  | Triglycerides in LDL-6 |
|  |  | Cholesterol in LDL-1 |
|  |  | Cholesterol in LDL-2 |
|  |  | Cholesterol in LDL-3 |
|  |  | Cholesterol in LDL-4 |
|  |  | Cholesterol in LDL-5 |
|  |  | Cholesterol in LDL-6 |
|  |  | Free Cholesterol in LDL-1 |
|  |  | Free Cholesterol in LDL-2 |
|  |  | Free Cholesterol in LDL-3 |
|  |  | Free Cholesterol in LDL-4 |
|  |  | Free Cholesterol in LDL-5 |
|  |  | Free Cholesterol in LDL-6 |
|  |  | Phospholipids in LDL-1 |
|  |  | Phospholipids in LDL-2 |
|  |  | Phospholipids in LDL-3 |
|  |  | Phospholipids in LDL-4 |
|  |  | Phospholipids in LDL-5 |
|  |  | Phospholipids in LDL-6 |
|  |  | Apolipoprotein B in LDL-1 |
|  |  | Apolipoprotein B in LDL-2 |
|  |  | Apolipoprotein B in LDL-3 |
|  |  | Apolipoprotein B in LDL-4 |
|  |  | Apolipoprotein B in LDL-5 |
|  |  | Apolipoprotein B in LDL-6 |
|  | Lipid Concentrations in HDL | Triglycerides in HDL |
|  |  | Free Cholesterol in HDL |
|  |  | Phospholipids in HDL |
|  |  | Apolipoprotein A1 in HDL |
|  |  | Apolipoprotein A2 in HDL |
|  | Lipid Concentrations in HDL Subfractions | Triglycerides in HDL-1 |
|  |  | Triglycerides in HDL-2 |
|  |  | Triglycerides in HDL-3 |
|  |  | Triglycerides in HDL-4 |
|  |  | Cholesterol in HDL-1 |
|  |  | Cholesterol in HDL-2 |
|  |  | Cholesterol in HDL-3 |
|  |  | Cholesterol in HDL-4 |
|  |  | Free Cholesterol in HDL-1 |
|  |  | Free Cholesterol in HDL-2 |
|  |  | Free Cholesterol in HDL-3 |
|  |  | Free Cholesterol in HDL-4 |
|  |  | Phospholipids in HDL-1 |
|  |  | Phospholipids in HDL-2 |
|  |  | Phospholipids in HDL-3 |
|  |  | Phospholipids in HDL-4 |
|  |  | Apolipoprotein A1 in HDL-1 |
|  |  | Apolipoprotein A1 in HDL-2 |
|  |  | Apolipoprotein A1 in HDL-3 |
|  |  | Apolipoprotein A1 in HDL-4 |
|  |  | Apolipoprotein A2 in HDL-1 |
|  |  | Apolipoprotein A2 in HDL-2 |
|  |  | Apolipoprotein A2 in HDL-3 |
|  |  | Apolipoprotein A2 in HDL-4 |
| The table lists all serum analytes quantified in this study. Soluble metabolites, quantified using the Bruker B.I.Quant-PS (version 2.0.0) panel, are grouped by major biochemical categories. Lipoprotein parameters include main clinical lipids, lipoprotein particle concentrations, and the lipid composition of various lipoprotein main fractions and their subfractions as determined by the Bruker B.I.LISA (version 1.0.0) panel, part of the Bruker IVDr platform. | | |

## Supplementary Table S2

Supplementary Table S2: Comparative analysis of metabolite associations with active metastasis from logistic regression models.

| **Metabolite** | **RFE Importance (Mean Decrease Gini)** | **OPLS-DA VIP (> 1)** | **RFE Model OR per SD (95% CI)** | **RFE Model P-Value** | **RFE Model BH-FDR** | **OPLS-DA Model OR per SD (95% CI)** | **OPLS-DA Model P-Value** | **OPLS-DA Model BH-FDR** |
| --- | --- | --- | --- | --- | --- | --- | --- | --- |
| **Pyruvate** | 15.65 | 2.24 | **1.91 (1.47 - 2.49)** | **<0.001** | **0.028** | **1.64 (1.30 - 2.07)** | **<0.001** | **0.013** |
| Glycine | 13.00 | - | 1.17 (0.90 - 1.54) | 0.239 | 0.634 | n.i. | n.i. | - |
| **Glucose** | 12.87 | 1.47 | **1.28 (1.06 - 1.55)** | **0.009** | 0.126 | 1.17 (0.98 - 1.40) | 0.083 | 0.216 |
| Alanine | 12.16 | - | 0.84 (0.62 - 1.13) | 0.249 | 0.634 | n.i. | n.i. | - |
| **Glutamate** | 11.98 | 1.28 | **1.32 (1.00 - 1.72)** | **0.046** | 0.182 | **1.30 (1.03 - 1.65)** | **0.028** | 0.091 |
| Lactate | 11.33 | 1.54 | 0.86 (0.65 - 1.13) | 0.277 | 0.646 | 0.83 (0.65 - 1.06) | 0.131 | 0.243 |
| Glutamine | 11.16 | 1.07 | 0.98 (0.76 - 1.27) | 0.890 | 0.923 | 0.92 (0.76 - 1.12) | 0.397 | 0.573 |
| Valine | 9.66 | - | 0.71 (0.50 - 1.00) | 0.052 | 0.182 | n.i. | n.i. | - |
| **Citrate** | 9.52 | - | **0.74 (0.57 - 0.97)** | **0.027** | 0.182 | n.i. | n.i. | - |
| Creatinine | 9.03 | - | 0.93 (0.74 - 1.16) | 0.500 | 0.787 | n.i. | n.i. | - |
| Leucine | 8.94 | - | 1.05 (0.77 - 1.42) | 0.759 | 0.923 | n.i. | n.i. | - |
| Glycerol | 8.71 | 1.19 | 0.98 (0.81 - 1.19) | 0.851 | 0.923 | 0.99 (0.82 - 1.19) | 0.925 | 0.925 |
| **Histidine** | 8.68 | 1.23 | **0.80 (0.64 - 0.99)** | **0.039** | 0.182 | **0.76 (0.62 - 0.92)** | **0.006** | **0.039** |
| Acetone | 7.94 | 1.33 | 0.99 (0.75 - 1.30) | 0.938 | 0.938 | 0.93 (0.72 - 1.20) | 0.553 | 0.719 |
| 3-Hydroxybutyrate | 7.88 | 1.47 | 0.83 (0.57 - 1.22) | 0.348 | 0.750 | 0.85 (0.59 - 1.21) | 0.359 | 0.573 |
| Lysine | 7.88 | - | 1.21 (0.94 - 1.55) | 0.132 | 0.411 | n.i. | n.i. | - |
| **Phenylalanine** | 7.38 | 1.22 | **1.40 (1.07 - 1.82)** | **0.014** | 0.131 | 1.20 (0.95 - 1.50) | 0.123 | 0.243 |
| Dimethylsulfone | 6.67 | - | 0.96 (0.70 - 1.32) | 0.791 | 0.923 | n.i. | n.i. | - |
| Isoleucine | 5.88 | - | 0.89 (0.64 - 1.24) | 0.503 | 0.787 | n.i. | n.i. | - |
| Tyrosine | 5.68 | - | 0.96 (0.73 - 1.26) | 0.773 | 0.923 | n.i. | n.i. | - |
| Methionine | 5.62 | - | 1.05 (0.81 - 1.36) | 0.690 | 0.923 | n.i. | n.i. | - |
| Creatine | 5.55 | - | 0.90 (0.69 - 1.16) | 0.419 | 0.787 | n.i. | n.i. | - |
| Proline | 5.35 | - | 0.94 (0.74 - 1.19) | 0.583 | 0.859 | n.i. | n.i. | - |
| Ornithine | 5.09 | - | 1.08 (0.87 - 1.34) | 0.506 | 0.787 | n.i. | n.i. | - |
| **Acetoacetate** | 5.08 | 1.30 | **1.36 (1.01 - 1.83)** | **0.041** | 0.182 | **1.42 (1.07 - 1.87)** | **0.014** | 0.061 |
| Acetate | 4.53 | - | 1.02 (0.83 - 1.25) | 0.861 | 0.923 | n.i. | n.i. | - |
| Formate | 3.38 | 1.08 | 1.08 (0.88 - 1.31) | 0.471 | 0.787 | 1.03 (0.85 - 1.25) | 0.746 | 0.875 |
| Succinate | 1.87 | 1.30 | 1.03 (0.78 - 1.35) | 0.845 | 0.923 | 1.03 (0.79 - 1.34) | 0.808 | 0.875 |
| Metabolites are listed based on their ranking by Random Forest Feature Importance (Mean Decrease Gini) from the Recursive Feature Elimination (RFE) analysis on the training set. 'RFE Model OR per SD (95% CI)' and 'RFE Model P-Value' represent the Odds Ratio per Standard Deviation (OR per SD), its 95% Confidence Interval (CI), and the P-value for the association with active metastasis from the logistic regression model trained on the 28 RFE-selected metabolites. 'OPLS-DA Model OR per SD (95% CI)' and 'OPLS-DA Model P-Value' present the corresponding values from the logistic regression model trained on the 13 OPLS-DA (VIP > 1) selected metabolites; '-' indicates the metabolite had a VIP score ≤ 1 or was not among the 13 selected by OPLS-DA for the logistic regression. The 'BH-FDR' columns show P-values adjusted for multiple testing using the Benjamini-Hochberg method. Values highlighted in bold meet the respective significance threshold (P-Value < 0.05 or BH-FDR < 0.05). | | | | | | | | |
| **Abbreviations: BH: Benjamini-Hochberg. FDR: False Discovery Rate. VIP: Variable Importance in Projection. RFE: Recursive Feature Elimination. OR: Odds Ratio. SD: Standard Deviation. CI: Confidence Interval.** | | | | | | | | |

## Supplementary Table S3

Supplementary Table S3: Descriptive statistics of serum metabolite concentrations in melanoma patients with and without active metastasis.

| **Metabolite** | **n (No AM)** | **Median (No AM)** | **IQR (No AM)** | **Mean (No AM)** | **SD (No AM)** | **n (AM)** | **Median (AM)** | **IQR (AM)** | **Mean (AM)** | **SD (AM)** | **p-value** |
| --- | --- | --- | --- | --- | --- | --- | --- | --- | --- | --- | --- |
| **Pyruvate** | 1469 | 0.07 | 0.06 - 0.1 | 0.08 | 0.04 | 229 | 0.09 | 0.07 - 0.12 | 0.10 | 0.05 | **<0.001** |
| Acetoacetate | 1469 | 0.00 | 0 - 0.01 | 0.01 | 0.02 | 229 | 0.00 | 0 - 0.01 | 0.01 | 0.03 | 0.054 |
| Glutamate | 1469 | 0.12 | 0.07 - 0.16 | 0.12 | 0.07 | 229 | 0.12 | 0.09 - 0.16 | 0.13 | 0.08 | 0.194 |
| **Histidine** | 1469 | 0.08 | 0.07 - 0.09 | 0.08 | 0.02 | 229 | 0.07 | 0.06 - 0.09 | 0.08 | 0.03 | **0.003** |
| **Phenylalanine** | 1469 | 0.06 | 0.05 - 0.08 | 0.07 | 0.02 | 229 | 0.07 | 0.06 - 0.08 | 0.07 | 0.02 | **<0.001** |
| **Glucose** | 1469 | 5.30 | 4.6 - 6.1 | 5.73 | 2.10 | 229 | 5.50 | 4.9 - 6.6 | 6.21 | 2.47 | **0.002** |
| Citrate | 1469 | 0.14 | 0.1 - 0.18 | 0.14 | 0.07 | 229 | 0.14 | 0.09 - 0.18 | 0.14 | 0.07 | 0.911 |
| Data are presented as n (number of samples), median (interquartile range, IQR), and mean (standard deviation, SD) for 7 selected metabolites on their original concentration scale (mmol/L). P-values were calculated using the Wilcoxon rank-sum test comparing patients with active metastasis to those without active metastasis. AM: Active Metastasis. | | | | | | | | | | | |

## Supplementary Table S4

Supplementary Table S4: Detailed results of subgroup-specific predictive models.

| **Subgroup Analysis** | **Patient Selection Criteria (Samples)** | **Model Training/Testing (Samples (Unique Patients))** | **Modelling Approach** | **AUC on Test Set (95% CI)** | **Metabolite** | **OR per SD (95% CI)** | **P-Value** | **BH-FDR** |
| --- | --- | --- | --- | --- | --- | --- | --- | --- |
| ICI Therapy vs. Other Systemic Therapies | Samples from patients receiving systemic therapy (N=320; ICI: n=171, Other: n=149) | Train: 187 (91 pat.), Test: 133 (60 pat.) | OPLS-DA based Logistic Regression | 0.704 (0.608 - 0.799) | Valine | 1.81 (0.96 - 3.42) | 0.065 | 0.173 |
|  |  |  |  |  | **Citrate** | **1.61 (1.10 - 2.35)** | **0.014** | 0.112 |
|  |  |  |  |  | Pyruvate | 1.32 (0.89 - 1.96) | 0.167 | 0.334 |
|  |  |  |  |  | Leucine | 1.24 (0.73 - 2.12) | 0.431 | 0.550 |
|  |  |  |  |  | Lysine | 1.22 (0.84 - 1.76) | 0.304 | 0.486 |
|  |  |  |  |  | Alanine | 1.17 (0.76 - 1.80) | 0.481 | 0.550 |
|  |  |  |  |  | Tyrosine | 0.91 (0.58 - 1.42) | 0.670 | 0.670 |
|  |  |  |  |  | **Isoleucine** | **0.59 (0.35 - 0.97)** | **0.038** | 0.152 |
|  |  |  | RFE based Logistic Regression | 0.721 (0.627 - 0.815) | **Citrate** | **1.55 (1.04 - 2.32)** | **0.031** | 0.217 |
|  |  |  |  |  | Valine | 1.38 (0.78 - 2.44) | 0.265 | 0.540 |
|  |  |  |  |  | Pyruvate | 1.29 (0.88 - 1.90) | 0.192 | 0.540 |
|  |  |  |  |  | Lysine | 1.20 (0.83 - 1.74) | 0.332 | 0.540 |
|  |  |  |  |  | Alanine | 1.13 (0.75 - 1.72) | 0.554 | 0.646 |
|  |  |  |  |  | Leucine | 1.04 (0.62 - 1.75) | 0.878 | 0.878 |
|  |  |  |  |  | Glycine | 0.85 (0.59 - 1.23) | 0.386 | 0.540 |
| Active Brain Met vs. Other Active Met Locations | Samples from patients with active metastatic disease (N=229; Brain Met: n=37, Other Met: n=192) | Train: 131 (84 pat.), Test: 98 (55 pat.) | OPLS-DA based Logistic Regression | 0.631 (0.507 - 0.754) | 3-Hydroxybutyrate | 2.21 (0.64 - 7.55) | 0.207 | 0.635 |
|  |  |  |  |  | Lysine | 1.65 (0.67 - 4.04) | 0.272 | 0.635 |
|  |  |  |  |  | Acetoacetate | 1.48 (0.49 - 4.49) | 0.492 | 0.837 |
|  |  |  |  |  | Histidine | 1.32 (0.65 - 2.67) | 0.442 | 0.837 |
|  |  |  |  |  | Glutamate | 1.26 (0.45 - 3.57) | 0.658 | 0.837 |
|  |  |  |  |  | Acetate | 1.09 (0.48 - 2.49) | 0.839 | 0.869 |
|  |  |  |  |  | Glutamine | 1.08 (0.47 - 2.46) | 0.855 | 0.869 |
|  |  |  |  |  | Phenylalanine | 0.92 (0.35 - 2.42) | 0.869 | 0.869 |
|  |  |  |  |  | Tyrosine | 0.79 (0.30 - 2.05) | 0.622 | 0.837 |
|  |  |  |  |  | Glucose | 0.75 (0.25 - 2.26) | 0.612 | 0.837 |
|  |  |  |  |  | Formate | 0.52 (0.19 - 1.39) | 0.190 | 0.635 |
|  |  |  |  |  | Succinate | 0.41 (0.09 - 1.87) | 0.251 | 0.635 |
|  |  |  |  |  | **Acetone** | **0.30 (0.09 - 1.00)** | **0.049** | 0.635 |
|  |  |  |  |  | Creatine | 0.16 (0.01 - 3.36) | 0.241 | 0.635 |
|  |  |  | RFE based Logistic Regression | 0.553 (0.439 - 0.668) | **Proline** | **0.50 (0.25 - 0.98)** | **0.045** | **0.045** |
| BRAF Mutation vs. Other (in Active Met with Mutation Analysis) | Samples from patients with active metastasis and mutation analysis (N=216; BRAF mutated: n=107, Other: n=109) | Train: 119 (78 pat.), Test: 97 (52 pat.) | OPLS-DA based Logistic Regression | 0.599 (0.485 - 0.714) | **Acetate** | **4.95 (1.50 - 16.35)** | **0.009** | 0.102 |
|  |  |  |  |  | Creatine | 1.58 (0.81 - 3.07) | 0.175 | 0.531 |
|  |  |  |  |  | Pyruvate | 1.29 (0.75 - 2.20) | 0.357 | 0.857 |
|  |  |  |  |  | Proline | 1.09 (0.63 - 1.89) | 0.768 | 0.864 |
|  |  |  |  |  | Tyrosine | 1.07 (0.65 - 1.78) | 0.792 | 0.864 |
|  |  |  |  |  | Threonine | 0.99 (0.61 - 1.62) | 0.982 | 0.982 |
|  |  |  |  |  | Glutamate | 0.90 (0.51 - 1.58) | 0.707 | 0.864 |
|  |  |  |  |  | Glycine | 0.88 (0.52 - 1.48) | 0.624 | 0.864 |
|  |  |  |  |  | Ornithine | 0.85 (0.53 - 1.37) | 0.504 | 0.864 |
|  |  |  |  |  | Alanine | 0.83 (0.46 - 1.49) | 0.533 | 0.864 |
|  |  |  |  |  | Glucose | 0.61 (0.29 - 1.25) | 0.177 | 0.531 |
|  |  |  |  |  | **Phenylalanine** | **0.38 (0.17 - 0.84)** | **0.017** | 0.102 |
|  |  |  | RFE based Logistic Regression | 0.655 (0.545 - 0.765) | **Acetate** | **3.16 (1.18 - 8.49)** | **0.022** | **0.044** |
|  |  |  |  |  | Alanine | 0.73 (0.49 - 1.09) | 0.124 | 0.124 |
| For each subgroup analysis, separate Orthogonal Partial Least Squares Discriminant Analysis (OPLS-DA) and Recursive Feature Elimination (RFE) based logistic regression models were trained and tested. This table provides the Area Under the Curve (AUC) with 95% Confidence Interval (CI) for each model on its respective test set, along with a list of all metabolites included in each final subgroup-specific logistic regression model. For each metabolite, the Odds Ratio (OR) per standard deviation, 95% CI, and the corresponding p-value are shown. The 'BH-FDR' column displays p-values adjusted for multiple comparisons within each specific subgroup model using the Benjamini-Hochberg method. Values highlighted in bold meet the respective significance threshold (P-Value < 0.05 & 95% CI for OR not including 1.0, or BH-FDR < 0.05). | | | | | | | | |
| **Abbreviations: BH, Benjamini-Hochberg; FDR, False Discovery Rate; ICI, Immune Checkpoint Inhibitor; Met, Metastases; OR, Odds Ratio; SD, Standard Deviation; CI, Confidence Interval.** | | | | | | | | |

## Supplementary Table S5

Supplementary Table S5. Comparison of baseline characteristics between samples with and without active metastasis.

| Characteristic | No Active Metastasis (n=1469) | Active Metastasis (n=229) | P-Value |
| --- | --- | --- | --- |
| **Age (years)** | 66.3 ± 14.6 | 64.8 ± 14.9 | 0.171 |
| **Gender** |  |  | 0.689 |
| Female | 608 (41.4%) | 91 (39.7%) |  |
| Male | 861 (58.6%) | 138 (60.3%) |  |
| **Tumor Stage** |  |  | < 0.001 |
| 0 | 1 (0.1%) | 0 (0.0%) |  |
| IA | 144 (9.8%) | 0 (0.0%) |  |
| IB | 432 (29.4%) | 0 (0.0%) |  |
| IIA | 138 (9.4%) | 0 (0.0%) |  |
| IIB | 87 (5.9%) | 0 (0.0%) |  |
| IIC | 47 (3.2%) | 0 (0.0%) |  |
| IIIA | 59 (4.0%) | 11 (4.8%) |  |
| IIIB | 112 (7.6%) | 15 (6.6%) |  |
| IIIC | 131 (8.9%) | 27 (11.8%) |  |
| IIID | 6 (0.4%) | 2 (0.9%) |  |
| IV | 267 (18.2%) | 166 (72.5%) |  |
| Unknown / Missing | 45 (3.1%) | 8 (3.5%) |  |
| Data are presented as Mean ± Standard Deviation (SD) for continuous variables and number (n) with percentage (%) for categorical variables. The entire cohort (1698 samples) was stratified by the primary endpoint 'Active Metastasis'. P-values were assessed using independent samples t-test for age and Chi-squared test for categorical variables to evaluate the comparability of the two analytical groups. | | | |

## Supplementary Table S6

Supplementary Table S5: Comparative analysis of metabolite associations with active metastasis from logistic regression models in the 'on-time' (≤120 min) cohort.

Validation Set Performance: OPLS-DA Model AUC = 0.67 (95% CI: 0.59-0.75) | RFE Model AUC = 0.60 (95% CI: 0.51-0.69)

| **Metabolite** | **RFE Importance (Mean Decrease Gini)** | **OPLS-DA VIP (> 1)** | **RFE Model OR per SD (95% CI)** | **RFE Model P-Value** | **RFE Model BH-FDR** | **OPLS-DA Model OR per SD (95% CI)** | **OPLS-DA Model P-Value** | **OPLS-DA Model BH-FDR** |
| --- | --- | --- | --- | --- | --- | --- | --- | --- |
| **pyruvate** | 8.72 | 2.36 | **1.66 (1.12 - 2.46)** | **0.011** | 0.064 | **1.60 (1.11 - 2.31)** | **0.012** | **0.035** |
| **glycine** | 8.71 | 1.20 | 1.31 (0.92 - 1.87) | 0.130 | 0.251 | **1.56 (1.16 - 2.10)** | **0.003** | **0.025** |
| **glucose** | 8.64 | 1.77 | **1.60 (1.24 - 2.06)** | **<0.001** | **0.029** | **1.41 (1.11 - 1.79)** | **0.005** | **0.025** |
| alanine | 8.19 | 1.04 | 0.78 (0.53 - 1.16) | 0.220 | 0.354 | 0.75 (0.54 - 1.02) | 0.067 | 0.096 |
| **glutamine** | 7.09 | - | **1.56 (1.04 - 2.34)** | **0.031** | 0.127 | n.i. | n.i. | - |
| **valine** | 6.92 | - | **0.60 (0.37 - 0.96)** | **0.035** | 0.127 | n.i. | n.i. | - |
| **phenylalanine** | 6.60 | 1.42 | **1.75 (1.23 - 2.48)** | **0.002** | **0.029** | **1.32 (1.01 - 1.72)** | **0.043** | 0.072 |
| lactate | 6.51 | 2.05 | 1.13 (0.76 - 1.68) | 0.552 | 0.728 | 1.06 (0.76 - 1.48) | 0.738 | 0.738 |
| leucine | 6.12 | - | 1.17 (0.77 - 1.77) | 0.469 | 0.648 | n.i. | n.i. | - |
| lysine | 5.77 | - | 1.36 (0.97 - 1.91) | 0.071 | 0.170 | n.i. | n.i. | - |
| creatinine | 5.67 | - | 0.88 (0.65 - 1.18) | 0.388 | 0.592 | n.i. | n.i. | - |
| **citrate** | 5.28 | 1.28 | **0.68 (0.47 - 0.99)** | **0.043** | 0.130 | **0.68 (0.50 - 0.92)** | **0.014** | **0.035** |
| dimethylsufone | 5.07 | - | 1.15 (0.94 - 1.41) | 0.163 | 0.295 | n.i. | n.i. | - |
| glutamate | 5.03 | - | 0.99 (0.72 - 1.37) | 0.961 | 0.961 | n.i. | n.i. | - |
| acetone | 4.75 | - | 0.72 (0.50 - 1.03) | 0.073 | 0.170 | n.i. | n.i. | - |
| **ornithine** | 4.66 | 1.58 | **1.32 (1.01 - 1.72)** | **0.045** | 0.130 | **1.28 (1.02 - 1.62)** | **0.035** | 0.070 |
| glycerol | 4.57 | - | 0.95 (0.72 - 1.26) | 0.711 | 0.859 | n.i. | n.i. | - |
| **x3_hydroxybutyrate** | 4.33 | - | **0.51 (0.28 - 0.93)** | **0.028** | 0.127 | n.i. | n.i. | - |
| histidine | 4.29 | 1.09 | 0.82 (0.61 - 1.10) | 0.185 | 0.316 | 0.85 (0.66 - 1.09) | 0.211 | 0.234 |
| isoleucine | 4.05 | - | 1.04 (0.68 - 1.61) | 0.845 | 0.942 | n.i. | n.i. | - |
| proline | 3.93 | - | 0.96 (0.70 - 1.30) | 0.773 | 0.897 | n.i. | n.i. | - |
| methionine | 3.68 | - | 0.87 (0.62 - 1.23) | 0.432 | 0.626 | n.i. | n.i. | - |
| acetate | 3.64 | - | 1.01 (0.80 - 1.28) | 0.942 | 0.961 | n.i. | n.i. | - |
| creatine | 3.44 | - | 0.92 (0.64 - 1.31) | 0.647 | 0.816 | n.i. | n.i. | - |
| tyrosine | 3.32 | - | 0.71 (0.49 - 1.05) | 0.084 | 0.174 | n.i. | n.i. | - |
| formate | 2.87 | 1.58 | 1.25 (0.98 - 1.61) | 0.076 | 0.170 | 1.21 (0.96 - 1.52) | 0.109 | 0.136 |
| **acetoacetate** | 2.63 | - | **2.26 (1.32 - 3.86)** | **0.003** | **0.029** | n.i. | n.i. | - |
| **sarcosine** | 1.38 | - | **1.46 (1.12 - 1.91)** | **0.005** | **0.036** | n.i. | n.i. | - |
| succinate | 0.66 | - | 0.98 (0.65 - 1.47) | 0.916 | 0.961 | n.i. | n.i. | - |
| *Metabolites are listed based on their ranking by Random Forest Feature Importance (Mean Decrease Gini) from the Recursive Feature Elimination (RFE) analysis on the training set for the respective cohort. Metabolites selected only by the OPLS-DA model are listed at the bottom. 'RFE Model OR per SD (95% CI)' and 'RFE Model P-Value' represent the Odds Ratio per Standard Deviation (OR per SD), its 95% Confidence Interval (CI), and the P-value for the association with active metastasis from the logistic regression model trained on all RFE-selected metabolites for this cohort. 'OPLS-DA Model OR per SD (95% CI)' and 'OPLS-DA Model P-Value' present the corresponding values from the logistic regression model trained on the OPLS-DA (VIP > 1) selected metabolites. '-' indicates the metabolite had a VIP score ≤ 1 or was not selected by OPLS-DA, or had no RFE importance score. 'n.i.' (not included) indicates the metabolite was not part of the respective model. VIP: Variable Importance in Projection. RFE: Recursive Feature Elimination. OR: Odds Ratio. SD: Standard Deviation. CI: Confidence Interval.* | | | | | | | | |

## Supplementary Table S7

Supplementary Table S7: Comparative analysis of metabolite associations with active metastasis from logistic regression models in the 'delayed' (>120 min) cohort.

Validation Set Performance: OPLS-DA Model AUC = 0.55 (95% CI: 0.44-0.66) | RFE Model AUC = 0.49 (95% CI: 0.38-0.59)

| **Metabolite** | **RFE Importance (Mean Decrease Gini)** | **OPLS-DA VIP (> 1)** | **RFE Model OR per SD (95% CI)** | **RFE Model P-Value** | **RFE Model BH-FDR** | **OPLS-DA Model OR per SD (95% CI)** | **OPLS-DA Model P-Value** | **OPLS-DA Model BH-FDR** |
| --- | --- | --- | --- | --- | --- | --- | --- | --- |
| **glycine** | 20.01 | - | **1.41 (1.01 - 1.97)** | **0.046** | 0.069 | n.i. | n.i. | - |
| **glutamine** | 19.97 | 1.31 | **0.64 (0.47 - 0.86)** | **0.003** | **0.009** | **0.74 (0.57 - 0.98)** | **0.033** | 0.068 |
| **pyruvate** | 17.70 | 2.93 | **1.51 (1.16 - 1.98)** | **0.002** | **0.009** | **1.83 (1.31 - 2.56)** | **<0.001** | **0.007** |
| **glutamate** | 16.93 | 1.76 | 1.19 (0.88 - 1.60) | 0.257 | 0.276 | **1.40 (1.02 - 1.94)** | **0.039** | 0.068 |
| acetone | 14.01 | 1.37 | 1.13 (0.91 - 1.41) | 0.276 | 0.276 | 1.16 (0.93 - 1.45) | 0.195 | 0.236 |
| **histidine** | 12.47 | 1.05 | **0.72 (0.53 - 0.98)** | **0.034** | 0.068 | **0.73 (0.54 - 0.98)** | **0.038** | 0.068 |
| glycerol | - | 1.18 | n.i. | n.i. | - | 0.88 (0.64 - 1.21) | 0.433 | 0.433 |
| lactate | - | 2.23 | n.i. | n.i. | - | 0.79 (0.56 - 1.13) | 0.202 | 0.236 |
| *Metabolites are listed based on their ranking by Random Forest Feature Importance (Mean Decrease Gini) from the Recursive Feature Elimination (RFE) analysis on the training set for the respective cohort. Metabolites selected only by the OPLS-DA model are listed at the bottom. 'RFE Model OR per SD (95% CI)' and 'RFE Model P-Value' represent the Odds Ratio per Standard Deviation (OR per SD), its 95% Confidence Interval (CI), and the P-value for the association with active metastasis from the logistic regression model trained on all RFE-selected metabolites for this cohort. 'OPLS-DA Model OR per SD (95% CI)' and 'OPLS-DA Model P-Value' present the corresponding values from the logistic regression model trained on the OPLS-DA (VIP > 1) selected metabolites. '-' indicates the metabolite had a VIP score ≤ 1 or was not selected by OPLS-DA, or had no RFE importance score. 'n.i.' (not included) indicates the metabolite was not part of the respective model. VIP: Variable Importance in Projection. RFE: Recursive Feature Elimination. OR: Odds Ratio. SD: Standard Deviation. CI: Confidence Interval.* | | | | | | | | |

## Supplementary Figure S1: Serum concentrations of key metabolites in melanoma patients with and without active metastasis.


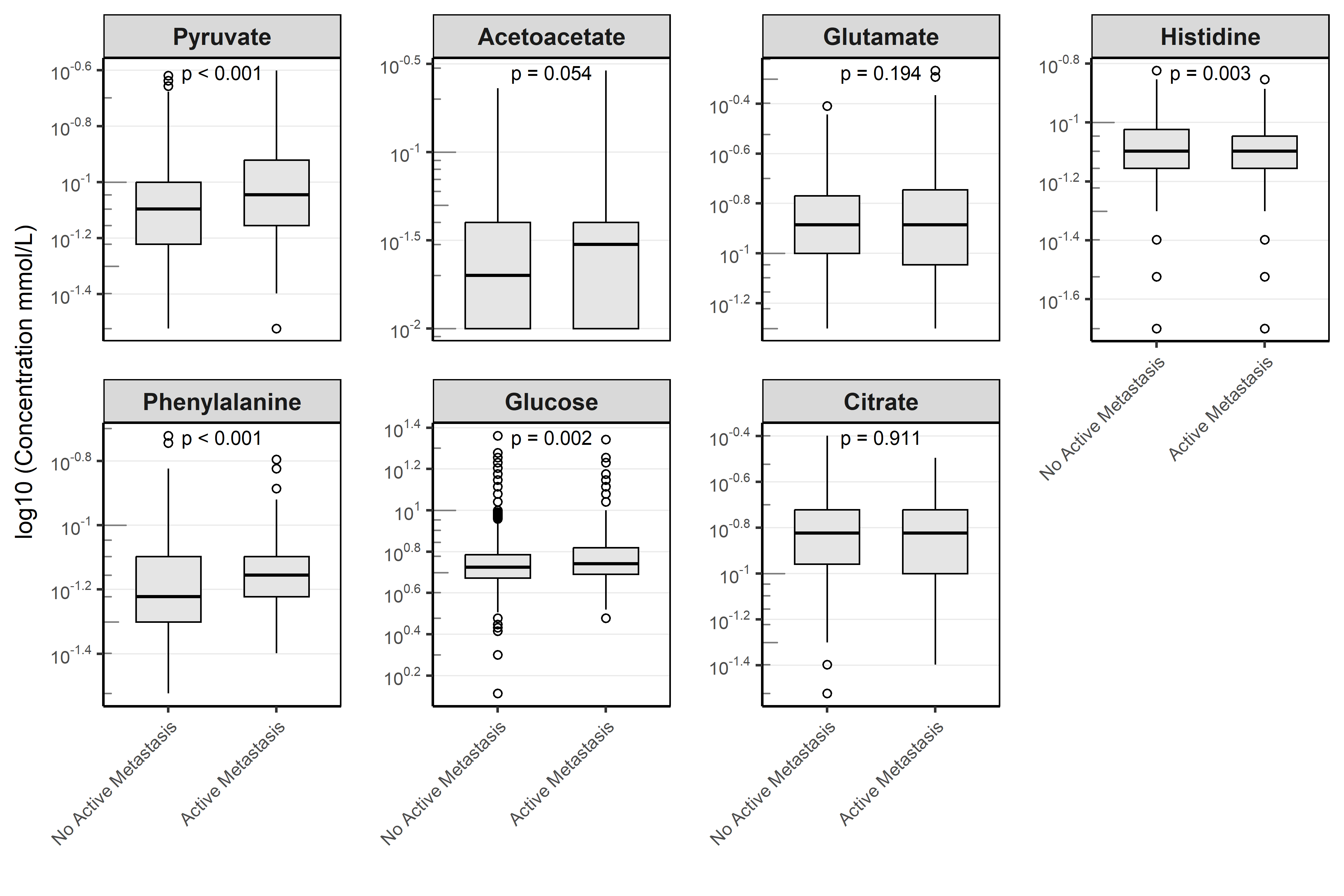


Boxplots illustrate the distribution of metabolite concentrations (log10-transformed, in mmol/L) for seven selected metabolites. Boxes represent the interquartile range (IQR) with the median shown as a horizontal line. Whiskers extend to 1.5 times the IQR from the box, and outliers beyond this range are shown as individual points. Statistical significance between groups (No Active Metastasis vs. Active Metastasis) was determined using the Wilcoxon rank-sum test; p-values are indicated above the respective comparisons. AM: Active Metastasis; No AM: No Active Metastasis.

## Supplementary Figure S2: Receiver Operating Characteristic (ROC) curves for subgroup-specific models evaluating test set performance.


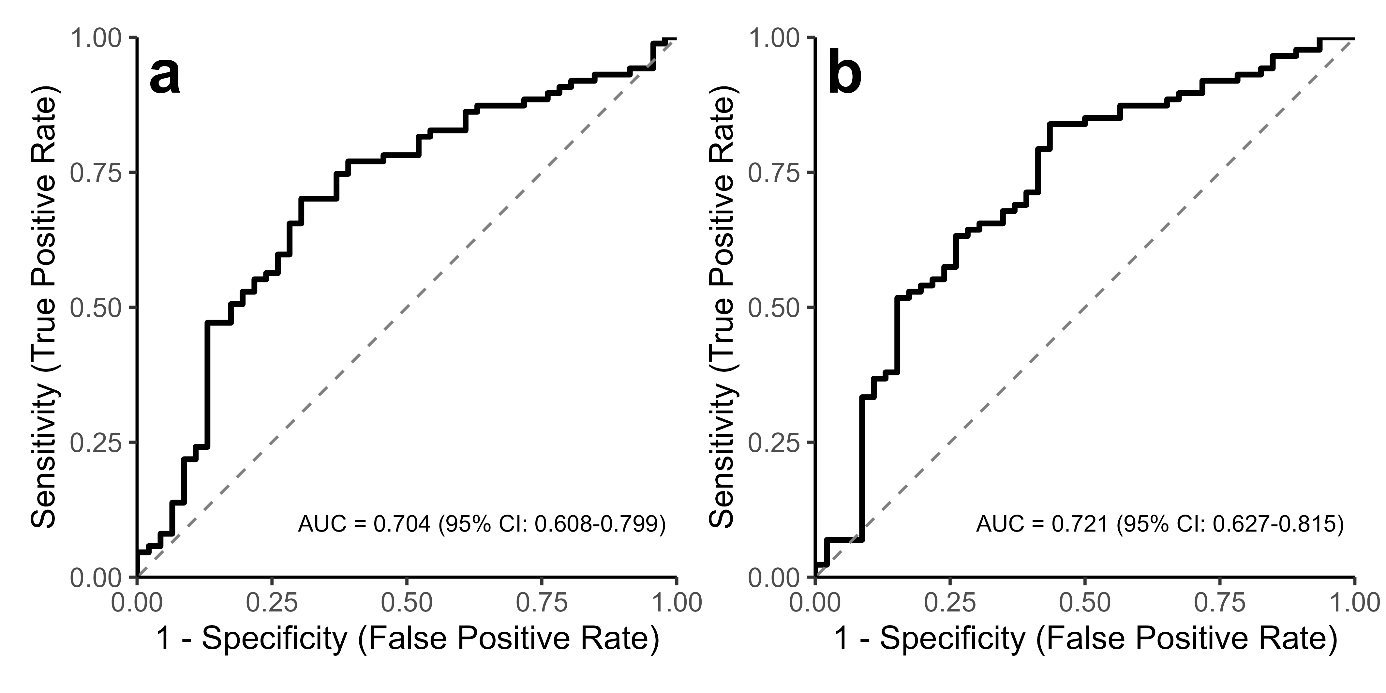


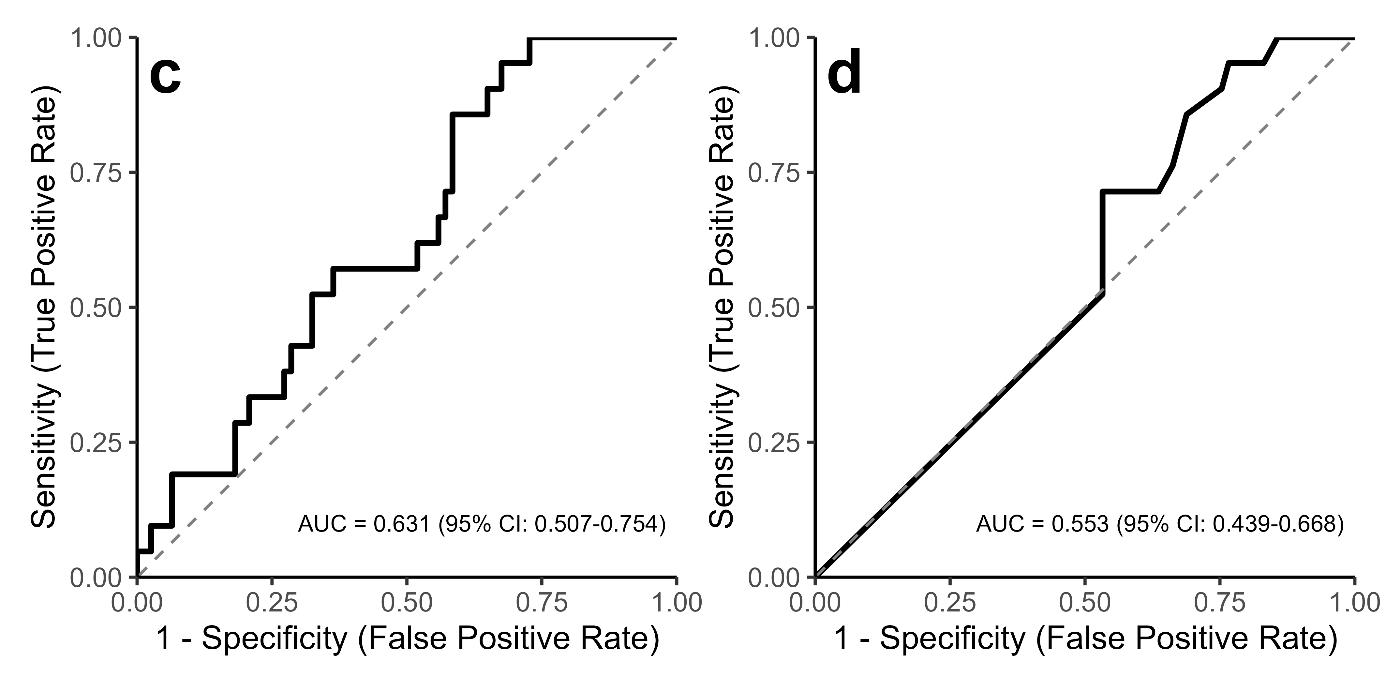


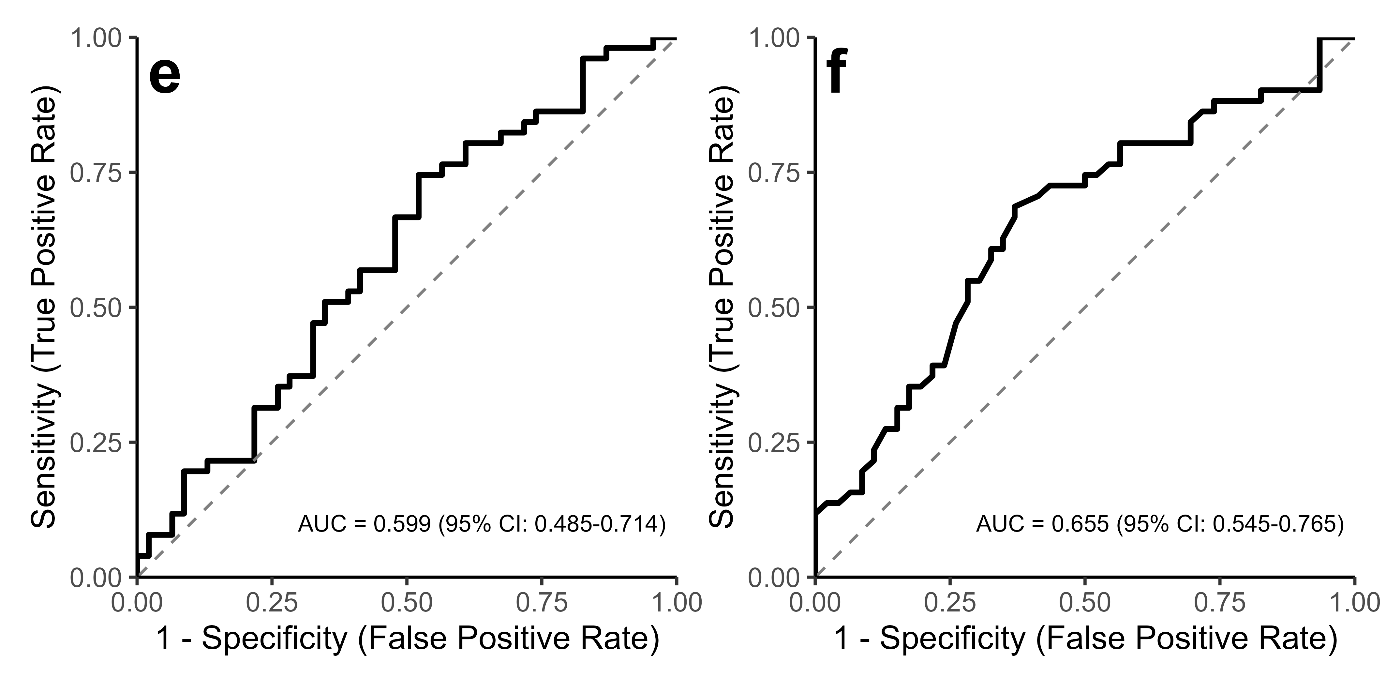


Models were trained to differentiate:
**(a, b)** Immune checkpoint inhibitor (ICI) therapy recipients vs. patients on other systemic therapies.
**(c, d)** Patients with active brain metastases vs. those with active metastases in other locations.
**(e, f)** Patients with BRAF-mutated vs. BRAF wild-type/other non-BRAF mutated tumors among patients with active metastasis.
Left column (a, c, e) displays ROC curves for OPLS-DA based logistic regression models. Right column (b, d, f) displays ROC curves for RFE based logistic regression models. AUC values and 95% Confidence Intervals (CI) are indicated for each model on its respective test set.

## Supplementary Figure S3: OPLS-DA Scores Plot


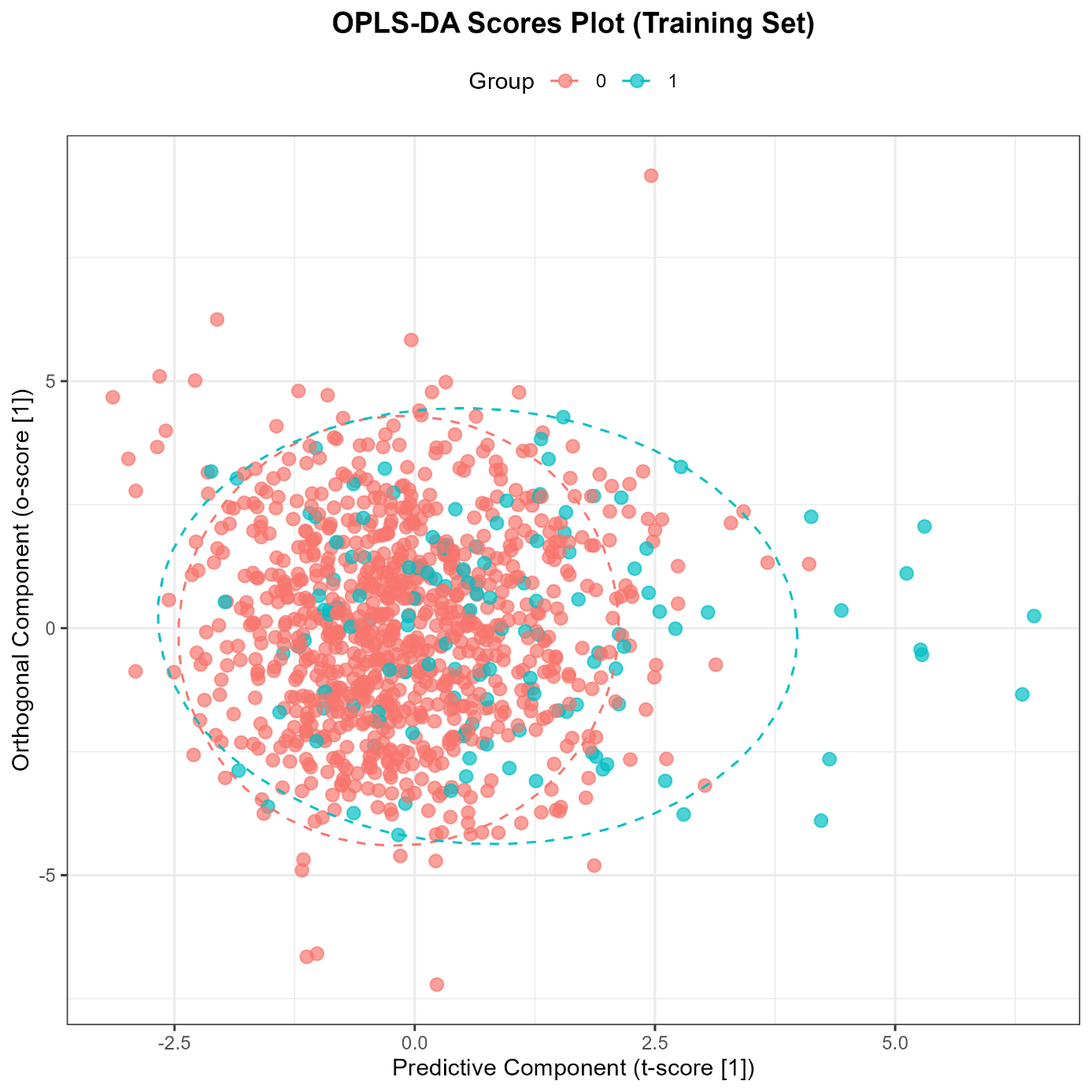


The scores plot from the Orthogonal Partial Least Squares Discriminant Analysis (OPLS-DA) model illustrates the metabolic separation between samples from patients with active metastasis (Yes) and without active metastasis (No) in the training set. Each point represents a single sample. The x-axis shows the predictive component (t-score), which captures the variation correlated with metastatic status. The y-axis shows the first orthogonal component (o-score), which represents structured variation not correlated with metastatic status. The ellipses represent the 95% confidence intervals for each group.
